# Deciphering the Role of p60AmotL2 in Epithelial Extrusion and Cell Detachment

**DOI:** 10.3390/cells12172158

**Published:** 2023-08-28

**Authors:** Weiyingqi Cui, Aravindh Subramani, Pedro Fonseca, Yumeng Zhang, Le Tong, Yuanyuan Zhang, Lars Egevad, Andreas Lundqvist, Lars Holmgren

**Affiliations:** Department of Oncology-Pathology, Bioclinicum J6:20, Solnavägen 30, Karolinska Institutet, 171 64 Stockholm, Swedenlars.egevad@ki.se (L.E.);

**Keywords:** apical cell extrusion, homeostasis, epithelial invasion, E-cadherin, mechanotransduction, cytoskeleton

## Abstract

Preserving an accurate cell count is crucial for maintaining homeostasis. Apical extrusion, a process in which redundant cells are eliminated by neighboring cells, plays a key role in this regard. Recent studies have revealed that apical extrusion can also be triggered in cells transformed by oncogenes, suggesting it may be a mechanism through which tumor cells escape their microenvironment. In previous work, we demonstrated that p60AmotL2 modulates the E-cadherin function by inhibiting its connection to radial actin filaments. This isoform of AmotL2 is expressed in invasive breast and colon tumors and promotes invasion in vitro and in vivo. Transcriptionally regulated by c-Fos, p60AmotL2 is induced by local stress signals such as severe hypoxia. In this study, we investigated the normal role of p60AmotL2 in epithelial tissues. We found that this isoform is predominantly expressed in the gut, where cells experience rapid turnover. Through time-lapse imaging, we present evidence that cells expressing p60AmotL2 are extruded by their normal neighboring cells. Based on these findings, we hypothesize that tumor cells exploit this pathway to detach from normal epithelia and invade surrounding tissues.

## 1. Introduction

Epithelial homeostasis, the critical balance ensuring proper structure and function of epithelial tissues, is maintained through the coordination of diverse cellular processes and factors [1]. Essential components, such as cell proliferation, differentiation, and apoptosis, work in tandem to replace damaged or aging cells, generate specialized cell types, and eliminate non-functional cells [2,3,4]. Moreover, cell-cell adhesion through tight junctions, adherens junctions, and desmosomes, as well as interactions with the extracellular matrix, reinforce the structural integrity and regulation of the epithelium [5,6,7]. Disruptions to this finely tuned balance can result in pathological conditions, such as chronic inflammation and cancer, highlighting the importance of understanding and preserving epithelial homeostasis.

Apical extrusion, a crucial mechanism for removing superfluous cells from the epithelial layer, involves the formation of an actomyosin contractile ring by adjacent cells, effectively forcing the aberrant cell out of the epithelial layer [8,9]. Pro-apoptotic signals produced by dying cells have been demonstrated to initiate apical extrusion in a caspase-dependent manner [10]. Recent research also suggests that the expulsion of live cells from an epithelial sheet contributes significantly to maintaining epithelial homeostasis, as exemplified by the rapid turnover of epithelial cells in mammalian gut villi [11,12]. Extruded live cells eventually undergo apoptosis or anoikis due to the lack of a conducive environment for continued proliferation [8]. However, defective cell extrusion plays a critical role in epithelial pathologies, such as cancer [13]. Cells with oncogenic transformations that are extruded exhibit increased survival signals, enabling them to bypass normal apoptotic processes and continue proliferating. The extrusion of transformed cells has been implicated in tumorigenesis and the metastasis of cancer cells from primary to distant sites. However, the molecular mechanisms behind this phenomenon are not yet fully understood.

E-cadherin, a key component of adherens junctions, plays an essential role in cell extrusion by maintaining cell-cell adhesion and regulating tissue homeostasis [14]. In the context of normal tissue turnover or cellular stress, E-cadherin’s functions are manipulated to permit the extrusion process. For example, in some cases, E-cadherin expression is downregulated or its localization altered, leading to a decrease in cell-cell adhesion. This downregulation can be induced by various pathways, such as through H-Ras overexpression leading to matrix metalloprotease-dependent cleavage, which weakens the adhesions between oncogenic and wild-type cells [15]. Consequently, this downregulation of E-cadherin promotes the extrusion of affected cells from the epithelial layer. Interestingly, E-cadherin’s role extends beyond the initial extrusion event. The loss of apical E-cadherin not only promotes cell extrusion but also activates other signaling molecules like Src at the preserved basolateral E-cadherin-containing junctions. This Src activation can lead to increased cell proliferation, contributing to the formation of cell clusters on the epithelial monolayer [16]. In certain contexts, the loss of E-cadherin in neighboring cells can lead to basal extrusion. This mechanism is particularly important in the context of cancerous transformations, where basal extrusion could potentially lead to the invasive behavior of cancer cells [17].

Angiomotin-like 2 (AmotL2) is a scaffold protein within the angiomotin protein family, playing an essential role in regulating cell junction formation, actin cytoskeleton organization, and maintenance of cell polarity [18,19,20,21,22]. The p100AmotL2 isoform is well-characterized and associated with the VE/E-cadherin/actin mechano-transductive complex [23,24]. In contrast, the p60AmotL2 isoform is significantly upregulated in invasive breast and colon cancers, acting as a negative regulator of apical-basal polarity by disrupting the connection between E-cadherin and radial actin filaments [25,26]. Activation of this isoform primarily occurs in response to stress signals such as severe hypoxia and is rarely detected in normal or tumor cells in vitro. In this study, we report that p60AmotL2 promotes apical extrusion and propose that tumor cells may exploit this process for delamination and invasion during cancer progression.

## 2. Materials and Methods

### 2.1. Antibodies and Reagents

The following primary antibodies were used: LDS-AmotL2 (polyclonal antibodies reactive to human AmotL2 C-terminal peptide and detecting both p60 and p100AmotL2 isoforms), E-cadherin (product number 610193, BD Biosciences, Franklin Lakes, NJ, USA), β-actin (product number ab3280, Abcam, Cambridge, UK), Caspase-3 (product number 9662S, Cell Signaling, Danvers, MA, USA), and cytokeratin (product number ab27988, Abcam, Cambridge, UK). The following secondary antibodies were utilized: Alexa 568 anti-mouse (product number A10037, Cytiva, Marlborough, MA, USA) and Alexa 488 anti-rabbit (product number A11008, Cytiva, Marlborough, MA, USA). Texas Red phalloidin (product number T7471, Life Technologies, Carlsbad, California, USA) and Alexa Fluor™ 647 Phalloidin (product number A22287, Invitrogen, Waltham, MA, USA) were employed to visualize actin filaments. Nuclei were stained with DAPI (product number F6057, Sigma-Aldrich, St. Louis, MO, USA). For western blot detection, the following reagents were used: ECL anti-mouse IgG horseradish peroxidase (product number NA931V, Cytiva, Marlborough, MA, USA) and ECL anti-rabbit IgG horseradish peroxidase (product number NA934V, Cytiva, Marlborough, MA, USA). In live cell imaging, nuclei were visualized using NucBlue™ Live ReadyProbes™ Reagent (product number R37605, Thermo Fisher Scientific, Waltham, MA, USA). Actin was stained with the SiR-actin Kit (product number CY-SC001, Spirochrome, Thurgau, Switzerland).

### 2.2. Immunohistochemistry of Patient Samples

The patient studies were approved by the Karolinska Hospital, Research Ethics Committee, Dnr 2006/1014-31/4, 2012/90-31/2 and 010/0066-32. The patient sample sections were subjected to deparaffinization using xylene, followed by rehydration through a series of ethanol solutions. To quench endogenous peroxidase activity, the slides were incubated with 3% hydrogen peroxide. For antigen retrieval, the slides were exposed to citrate buffer (pH 6.0) in a microwave oven for 20 min, followed by a cooling period of 20 min at room temperature. To mitigate nonspecific background staining, the slides were treated with horse serum as a blocking agent. Subsequently, the slides were subjected to an overnight incubation at 4 °C with a polyclonal antibody against AmotL2 (LDS-AmotL2, dilution 1:400). Following the incubation, the slides were thoroughly rinsed and then exposed to biotinylated horse anti-mouse secondary antibodies. This was succeeded by another round of rinsing and incubation, during which avidin-biotin-peroxidase complexes were applied to the slides. For visualization of immunostaining, the slides were immersed in a solution containing 0.05% 3,3′-diaminobenzidine tetrahydrochloride. Subsequently, counterstaining was performed using hematoxylin.

### 2.3. Mouse Experiments

Animal experiments were conducted in compliance with the Stockholm South Ethical Committee in Sweden (N129/15). All experiments utilized wild-type mice. Intestines were harvested at the animal facility at KMB, Karolinska Institutet, and processed separately to isolate the epithelial layer in the lab. All methods adhered to relevant guidelines and regulations, with experimental protocols approved by the Swedish Board of Agriculture.

### 2.4. Immunohistochemistry

Mouse small intestine samples were fixed in 4% paraformaldehyde (PFA; product number sc-281692, Santa Cruz Biotechnology, Santa Cruz, CA, USA) in 1×PBS and embedded into a cryomold. Tissues were then cut into 10-μm sections and mounted onto glass slides. The slides were rinsed once in 1×PBS, permeabilized with 0.5% TritonX-100 (product number X100, Sigma-Aldrich, St. Louis, MO, USA) for 15 min, and non-specific staining was blocked with 0.5% horse serum diluted in 1×PBS. The slides were incubated with primary antibodies overnight at 4 °C, washed 3 times for 10 min in 1×PBS, and positive signals were detected with a secondary antibody at room temperature for 1 h. The slides were washed 3 times for 10 min in 1×PBS and mounted with DAPI-containing mounting media (product number F6182, Sigma-Aldrich, St. Louis, MO, USA). Immunohistochemistry of AmotL2 in human tissues was performed in collaboration with http://www.proteinatlas.org (accessed on 15 January 2021). The antibodies were generated in the Holmgren lab, and the results have not yet been made publicly available.

### 2.5. Western Blot (WB)

For cell lysate preparation, cells were treated with a lysis buffer that contained 50 mM Hepes buffer, 150 mM NaCl, 1.5 mM MgCl_2_, 1 mM EGTA, 10% glycerol, and 1% TritonX-100, along with a freshly added 1× protease inhibitor (product number 04693159001, Roche, Basel, Switzerland). This process was performed on ice. Following this, the mixture was subjected to centrifugation at 15,000 rpm for a span of 3 min and the supernatant was collected. Lysates were then combined with an SDS sample buffer (4×, product number 1225644, Novex, Wadsworth, OH, USA) that was enhanced with a 10% sample-reducing agent (product number 1176192, Novex). The proteins present were fractionated using a Bis-Tris precast polyacrylamide gel with a gradient of 4–12% (product number NP0322BOX, Novex, Wadsworth, OH, USA). These fractionated proteins were then moved onto a nitrocellulose membrane (product number 10401396, Whatman, Maidstone, UK). To block any non-specific binding, the membrane was treated with a solution of 5% non-fat milk and 0.1% Tween 20 in 1×PBS for an hour at room temperature. Following this, the membrane was incubated overnight at 4 °C with the primary antibody and was then subjected to treatment with the secondary antibody for an additional hour at room temperature. Finally, proteins that were labeled with antibodies were identified using a chemiluminescent substrate (ECL; product number RPN2232, Amersham Biosciences, Amersham, UK) using an iBright imaging system (Thermo Fisher Scientific, Waltham, MA, USA).

### 2.6. Western Blot Analysis of Mouse Small Intestine

Small intestine samples were collected from wild-type c57B6/J mice (*n* = 4), and intestinal villi and crypts were gently scraped using a cell scraper. The villi and crypts were homogenized in lysis buffer and heated at 95 °C for 5 min. Cell extracts were centrifuged at 15,000 rpm for 10 min, and the supernatants were collected for western blot analysis. The 25 μg of protein were loaded on a 10% Tris-Acetate gel at 120 V for 120 min. Detection was performed using a standard ECL kit. AmotL2 protein levels are presented relative to the beta-actin loading control.

### 2.7. Mouse Small Intestine Organoids

Mouse small intestine organoids were harvested and cultured following previously described methods [27]. In brief, small intestine tissue from sacrificed mice was sectioned into small fragments and rinsed thoroughly with cold 1×PBS until the supernatant remained clear. Tissue fragments containing crypts were isolated using Gentle Cell Dissociation Reagent (product number 07174, STEMCELL, Vancouver, BC, Canada) and filtered through 70 μm cell strainers. The fractions were embedded in Geltrex matrigel (product number A1413202, Thermo Fisher Scientific, Waltham, MA, USA) and maintained in IntestiCult™ Organoid Growth Medium (product number 06005, STEMCELL, Vancouver, BC, Canada) for the duration of the experiments.

### 2.8. Cell Culture

Madin-Darby Canine Kidney (MDCK) wild-type cells were obtained from the American Type Culture Collection (ATCC, Manassas, VA, USA). MDCK cells stably expressing E-cadherin tagged with a red fluorescent protein (MDCK-E-cad-RFP) were generously provided by Prof. Christopher P. Torret’s lab. All cell lines were cultured in Dulbecco’s Modified Eagle Medium (DMEM) (product number 31053028, Thermo Fisher Scientific, Waltham, MA, USA) supplemented with 10% fetal bovine serum (FBS, product number 16000044, Thermo Fisher Scientific, Waltham, MA, USA) and penicillin/streptomycin (product number 10378016, Thermo Fisher Scientific, Waltham, MA, USA).

MDCK cell lines stably expressing doxycycline-inducible p60AmotL2 were generated using the Gateway™ system (Thermo Fisher Scientific, Waltham, MA, USA), as previously described [25]. To induce p60AmotL2 expression, doxycycline (Dox) (10 ng/mL, product number D3447, Sigma-Aldrich, St. Louis, MO, USA) was added to the culture after seeding the cells. For plasmid transfections, MDCK cells were seeded on glass slides (BD Falcon Culture slides, BD Biosciences, Franklin Lakes, NJ, USA) in a growth medium one day prior to transfection. Plasmids p60AmotL2-GFP, p60AmotL2ΔILI-GFP, and myristoylated-GFP were transfected into the cells using the GenJet™ In Vitro DNA transfection reagent for MDCK cells (product number SL100489-MDCK, SignaGen Laboratories, Frederick, MD, USA), following the manufacturer’s protocol.

### 2.9. Immunofluorescent Staining

MDCK cells were cultured in 8-well chamber slides to confluency and fixed with 4% paraformaldehyde in PBS (product number sc-281692, Santa Cruz Biotechnology, Santa Cruz, CA, USA) for 10 min, permeabilized for 5 min with 0.5% TritonX-100 (product number X100, Sigma-Aldrich, St. Louis, MO, USA) in 1×PBS, washed three times with 1×PBS, and blocked with 5% horse serum in 1×PBS for 1 h before incubating with primary antibodies. After washing 3 times in 1×PBS, the slides were incubated with secondary antibodies diluted in 5% horse serum in 1×PBS. Following incubation with the secondary antibody, the slides were washed 3 times in 1×PBS and then mounted onto coverslips.

### 2.10. Overcrowding Assay

MDCK cells were plated at a concentration of 500,000 cells per cm^2^ and grown to confluency for 4 days in 8-well chamber slides. The slides were then fixed in 4% PFA (product number sc-281692, Santa Cruz Biotechnology, Santa Cruz, CA, USA) and subjected to immunofluorescence staining as described above in the cell immunostaining method. Slides were imaged using a Zeiss AxioObserver with LSM700 confocal module and a 63× objective (Zeiss, Oberkochen, Germany).

### 2.11. Co-immunoprecipitation(co-IP)

For the co-immunoprecipitation (co-IP) experiment, cells were rinsed with cold PBS and directly scraped off a 10 cm dish into a lysis buffer. The lysis buffer consisted of 50 mM Hepes buffer, 150 mM NaCl, 1.5 mM MgCl_2_, 1 mM EGTA, 10% glycerol, and 1% TritonX-100. The lysates were then centrifuged at 15,000 rpm for 5 min to remove debris. To minimize non-specific binding to Sepharose beads, cell lysates were pre-cleared by incubating with protein G Sepharose beads (product number ab193259, Abcam, Cambridge, UK) for 1.5 h at 4 °C. After pre-clearing, 2 µg of AmotL2 or control antibodies from the same species were added to the lysates and incubated overnight at 4 °C. Immunocomplexes were precipitated by adding protein G beads to the lysate and incubating for 2 h at 4 °C. The beads were then washed five times by centrifugation at 12,000 rpm for 20 s each time with the lysis buffer to remove non-specifically bound proteins. Finally, the protein-bead complexes were denatured, and the proteins were separated from the beads for analysis by western blotting.

### 2.12. Confocal Time-Lapse Imaging

MDCK RFP-E-Cadherin cells were seeded at confluency on a 2 mg/mL collagen matrix in a 35 mm Petri dish (Greiner bio-one, Kremsmünster, Germany). Cells were transfected with p60AmotL2-GFP, p60AmotL2ΔILI-GFP plasmid, or GFP-myristylated for 8h or more before obtaining confocal images. NucBlue™ Live ReadyProbes™ Reagent (product number R37605, Thermo Fisher Scientific, Waltham, MA, USA) was used to visualize nuclei. Images were captured using a Zeiss AxioObserver with LSM700 confocal module in 40× or 63× objective (Zeiss, Oberkochen, Germany). During the experiments, Petri dishes were maintained in a confocal integrated incubator (37 °C, 5% CO_2_, and a humid atmosphere). Images were captured every 30 min for at least 12 h. Image and video captures were processed and analyzed using the IMARIS software (https://imaris.oxinst.com (accessed on 14 January 2019)) and ImageJ software (version 1.53t).

### 2.13. Time-Lapse Imaging Using Incucyte^®^

MDCK wild-type cells were seeded in a 48-well plate (Thermo Fisher Scientific, Waltham, MA, USA) and allowed to reach 70% confluency. Subsequently, the cells were transfected with p60AmotL2-GFP or GFP-ctrl plasmid using the GenJet™ In Vitro DNA transfection reagent, as previously mentioned. Following transfection, the cells were transferred to the Incucyte^®^ Live-Cell Analysis System (Essen Bioscience, Ann Arbor, MI, USA). Live images were captured at 2-h intervals over a 48-h period using bright field and GFP imaging with 20× objective lenses. The imaging process was performed inside an incubator. To analyze the extruding cell numbers, Incucyte^®^ analysis software (Version 2021B, Essen Bioscience, Ann Arbor, MI, USA) was used.

### 2.14. Foci Formation Assay

MDCK wild-type cells and MDCK cells with Dox-inducible p60AmotL2 expression were mixed at a ratio of 50:1 and seeded on bovine collagen conjugated with Oregon Green (product number O10241, Thermo Fisher Scientific, Waltham, MA, USA) at a final concentration of 30 µg/mL. The cells were initially treated with Dox for 24 h to induce p60AmotL2 expression. Subsequently, they were treated with Hepatocyte Growth Factor (HGF, product number PHG0254, Thermo Fisher Scientific, Waltham, MA, USA) for the following 3 days. The cells, along with the collagen matrix, were then fixed and stained with LDS-AmotL2 antibody, as previously described. The following secondary antibodies were used: Alexa 568 anti-rabbit (product number A11011, Cytiva, Marlborough, MA, USA). Nuclei were stained with DAPI (product number F6057, Sigma-Aldrich, St. Louis, MO, USA), and Alexa Fluor™ 647 Phalloidin (product number A22287, Invitrogen, Waltham, MA, USA) was utilized for F-actin staining. The images were captured using an LSM700 confocal module with a 40× objective. Manual counting of foci and analysis using ImageJ software were performed to determine the results.

### 2.15. Proximity-Dependent Biotin Identification (BioID)

BioID plasmids were constructed using a mammalian gene expression lentiviral vector by combining cDNA fragments encoding human p100AmotL2 (Accession No: NM_001278683) with the N-terminus of E. coli biotin ligase (BirA). An empty vector with the same backbone was used as a negative control. All constructs were verified through restriction enzyme digestion. The p100AmotL2 BioID construct, and empty vector were packaged into lentivirus using Lipofectamine 3000 Transfection Reagent according to the manufacturer’s protocol. MDCK cells were transduced with the lentiviral constructs, and stable cell lines were generated using a 0.5 mg/mL geneticin selection. Stably transduced cells were cultured in DMEM medium, supplemented with 10% FBS and 0.5 mg/mL geneticin.

For the BioID experiment, transfected MDCK cells were treated with 50 µM biotin for 16 h, followed by harvesting in lysis buffer containing 50 mM Tris·HCl (pH 7.4), 8 M urea, 1 mM DTT, and protease inhibitors. Lysates were supplemented with 1% Triton X-100 before sonication. Biotinylated proteins were purified using streptavidin beads overnight at 4 °C. After washing five times with 8M urea in 50 mM Tris·HCl (pH 7.4) and once with 50 mM Tris·HCl (pH 7.4), the beads were resuspended in PBS and prepared for further protein analysis (Western Blotting and Mass Spectrometry analysis). Three independent experiments were performed (*n* = 3 in all groups), including ‘p100AmotL2 ± biotin’, and ‘empty vector ± biotin’.

Protein identification criteria for the BirA p100AmotL2 BioID construct were as follows: (1) at least two of three samples in the ‘p100AmotL2 + biotin’ group had a positive value; (2) the minimum value of the ‘p100AmotL2 + biotin’ group was equal to or higher than the maximum value of the ‘empty vector ± biotin’ and ‘p100AmotL2—biotin’ groups; (3) the average value of the ‘p100AmotL2 + biotin’ group was higher than those in the ‘empty vector ± biotin’ and ‘p100AmotL2—biotin’ groups.

## 3. Results

### 3.1. Expression of AmotL2 in Normal and Cancerous Tissues

**Figure 1:**
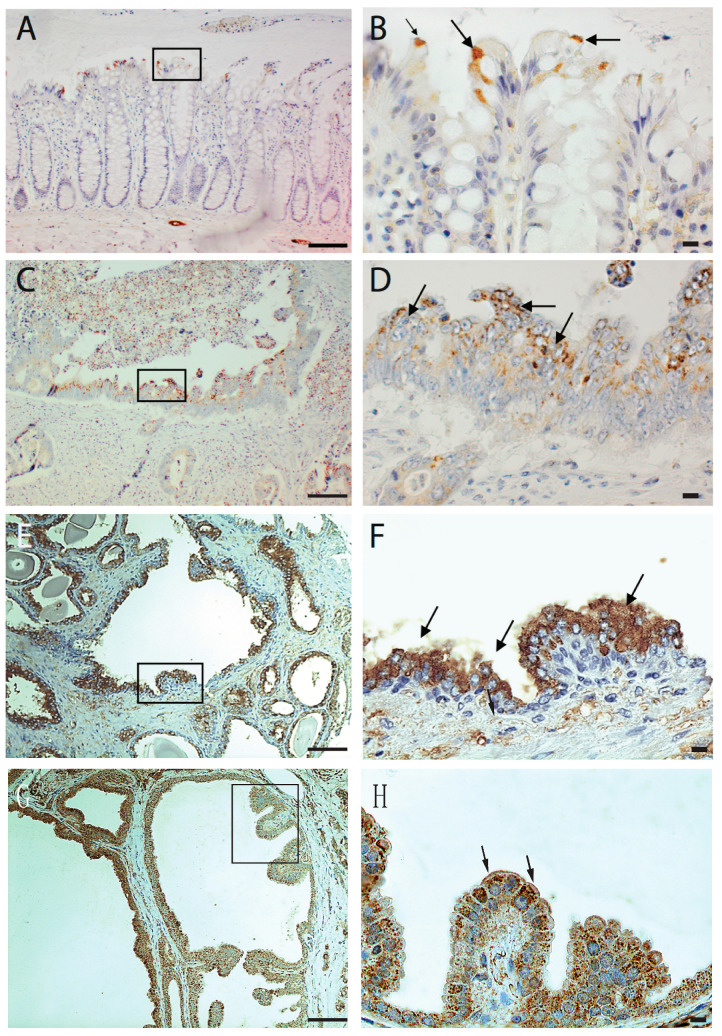
Immunohistochemical analysis of AmotL2 expression in human tissues. Human tissue paraffin sections were stained with immuno-affinity purified AmotL2 antibody (LDS- Human tissue paraffin sections were subjected to immuno-affinity staining using purified AmotL2 antibody (LDS-AmotL2). Positive AmotL2 staining is depicted in brown, while nuclei are counterstained with hematoxylin (blue). Regions of interest are highlighted by black squares, with corresponding magnifications presented in the left panel. Positive stained regions are indicated by arrows. (**A**,**B**) Normal human colon displaying positive AmotL2 signal, particularly in extruding epithelial cells. (**C**,**D**) Colon cancer (**E**,**F**) Prostate cancer and (**G**,**H**) Breast cancer specimens revealing altered AmotL2 expression. Left scale bar = 100 μm and right scale bar = 10 μm.

In our previous research, we have shown that p60AmotL2 is expressed by invasive cancers, including budding cells of colorectal cancer, infiltrative breast cancer, glioblastoma, and prostate and neuroendocrine cancers, while the corresponding normal tissue was negative. Notably, in colorectal cancer, p60AmotL2 expression was found to correlate with tumor stage and poor prognosis. In this study, we analyzed the expression of AmotL2 in normal colon tissue in greater detail by performing immunohistochemical staining on samples resected from colon cancer patients. Interestingly, positive staining was detected in specific colon epithelial cells undergoing extrusion into the colon lumen (Figure 1A,B). Further examination of glandular tumors, such as colon, prostate, and breast cancer, revealed p60AmotL2 expression specifically in tumor cells growing or being shed into the lumen of glandular structures (Figure 1C–H).

### 3.2. p60AmotL2 Is Expressed in Areas of Cell Shedding

**Figure 2:**
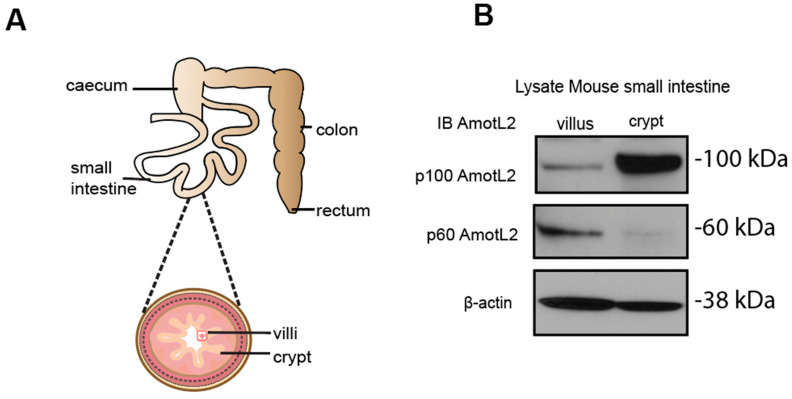

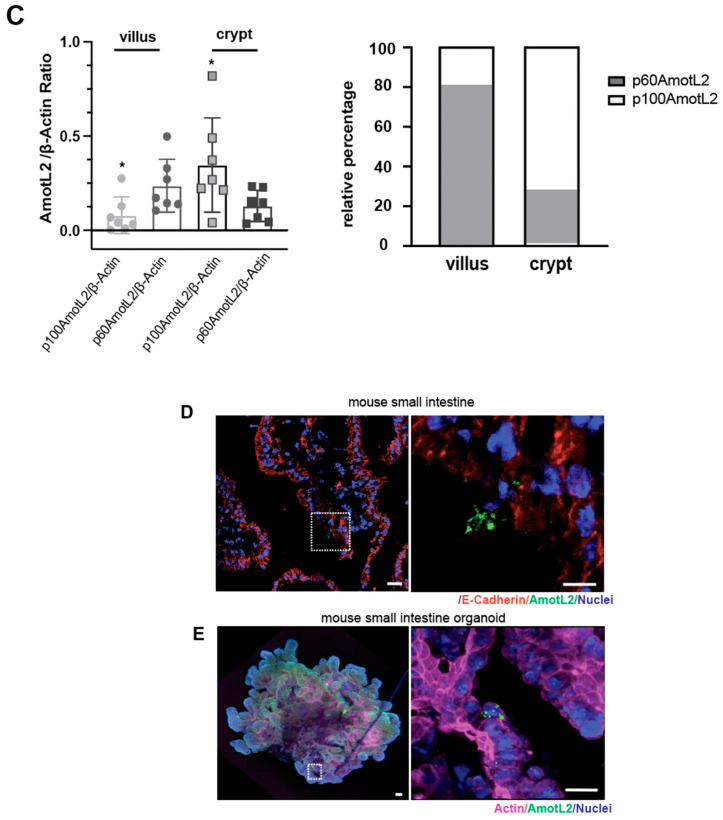
Expression and localization of AmotL2 in mouse intestine. (**A**) Schematic representation of the mouse gastrointestinal tract, showing crypt and villous tissues that were extracted and analyzed. (**B**) Western blot analysis of AmotL2 isoform expression in mouse small intestine fractions (villi and crypt). (**C**) Quantification of AmotL2 expression in the mouse small intestine was performed using western blot analysis. The left panel shows the ratio of p100 and p60AmotL2 to ßactin, represented by individual circles or squares corresponding to values obtained from each animal sample (*t*-test, * *p* < 0.05). The right panel presents a bar graph illustrating the relative expression levels of p100 and p60AmotL2. (**D**) Representative immunostainings of the mouse small intestine are shown. White squares indicate regions of interest. Scale bars in both the left and right panels represent 10 μm. (**E**) Immunostainings of organoids derived from the mouse small intestine. The arrow in the right panel indicates the presence of an extruding p60AmotL2-positive cell. Scale bars in both the left and right panels represent 10 μm. Data are mean ± SD from three independent experiments.

Given the localization of AmotL2 to areas of cell shedding in both normal and tumor tissues, we hypothesized that p60AmotL2 could be involved in the process of apical extrusion. We, therefore, analyzed whether p60AmotL2 was expressed in areas characterized by high turnover of cells and apical extrusion, such as the small intestine of the mouse. In the small intestine, proliferation is active in the stem cells localized to crypts. Epithelial cells migrate from a basal to an apical position and are eventually shed at the tip of the intestinal villi [28]. We mechanically separated the base of the villi (including the crypts) from the tip areas and analyzed the lysates by western blot (Figure 2A,B). Our data indicated that p60AmotL2 was primarily expressed in the villus fraction with lower expression in the crypt fraction. Conversely, p100AmotL2 was predominantly expressed in the crypt fraction (Figure 2B). Quantification of individual AmotL2 isoform expression and the relative expression of the two isoforms are shown in the bar diagrams in (Figure 2C). In addition, immunofluorescent staining of AmotL2 expression in the mouse small intestine showed a distinct signal in cells that were shed into the lumen (Figure 2D). A similar pattern was also observed in organoids derived from the mouse small intestine (Figure 2E).

### 3.3. Correlation between p60AmotL2 Expression and Overcrowding-Induced Apical Extrusion

**Figure 3:**
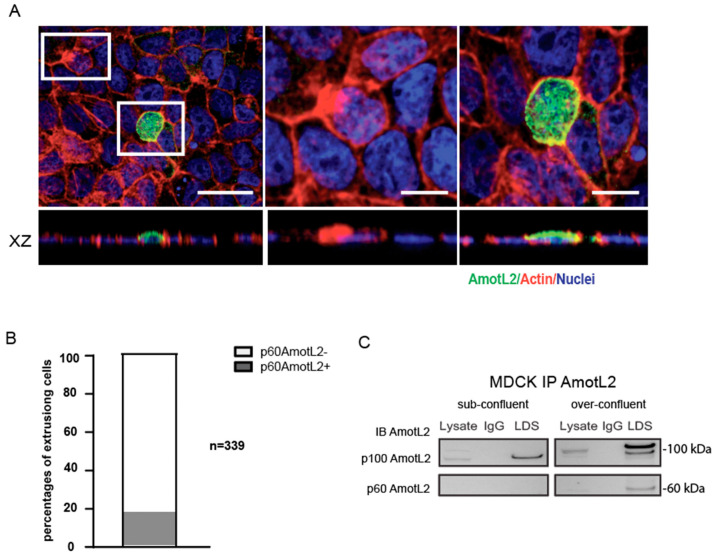
p60AmotL2 is induced in over-confluent MDCK cultures. (**A**) Immunofluorescent staining with LDS-AmotL2 (green) in over-confluent MDCK cells. Actin is visualized by phalloidin (red) and nuclei by DAPI (blue) staining. White squares highlight AmotL2-negative and -positive extruding cells, shown in higher magnification on the right panels. Scale bar left panel = 20 µm, middle and right panel =10 µm. (**B**) Quantification of the percentage of AmotL2-positive cells undergoing apical extrusion (*n* = 339, Mann–Whitney U-test, *p* < 0.001). (**C**) Western blot analysis of p100 and p60AmotL2 isoforms in sub-confluent and over-confluent MDCK cells. Data are mean ± SD from three independent experiments.

Previous studies have demonstrated that overcrowding of epithelial cells in culture activates cell extrusion to maintain a constant cell number [8,11]. In this study, we sought to examine the relationship between overcrowding-induced apical extrusion and p60AmotL2 expression. For this purpose, MDCK cells were used as a well-established model for studying epithelial biology and mechanisms of apical extrusion. Upon subjecting these cells to overcrowding conditions, the immunofluorescent analysis showed vesicular AmotL2 expression in extruding cells as characterized by the typical rosette formation of the neighboring cells (Figure 3A). Quantification of immunofluorescent stainings revealed that 35% of the extruding cells were positive for vesicular AmotL2 (Figure 3B). This vesicular pattern of AmotL2 is characteristic of the expression of the p60AmotL2 isoform, and the induction of p60AmotL2 expression by overcrowding was further verified by immunoprecipitation experiments (Figure 3C).

### 3.4. Establishing Causality between p60AmotL2 Expression and Apical Cell Extrusion

**Figure 4:**
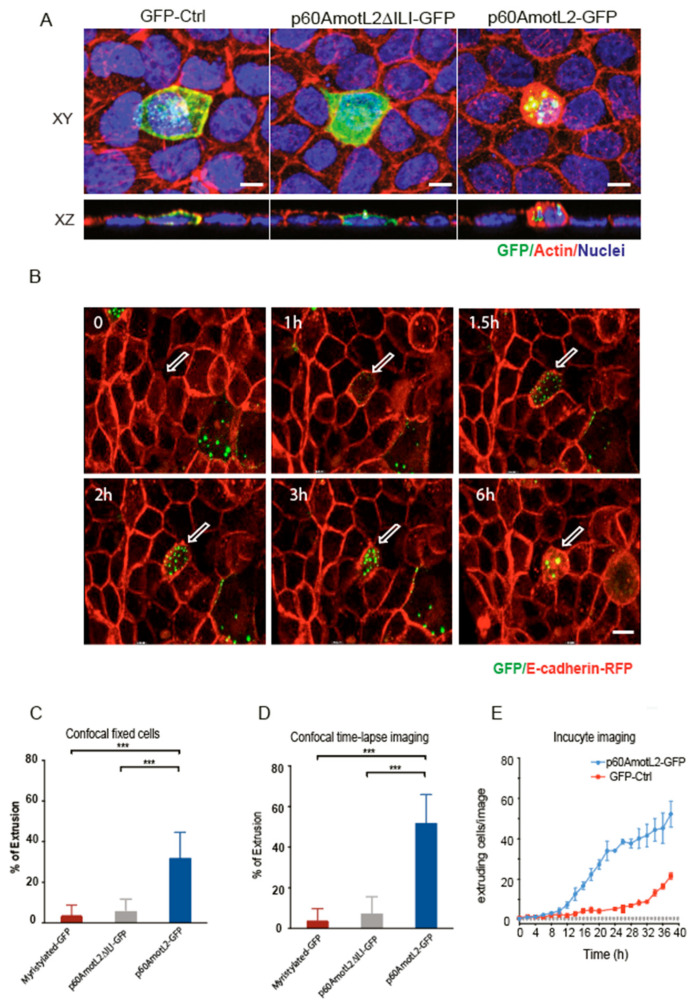
p60AmotL2 expression induces apical extrusion. (**A**) Immunofluorescent staining of MDCK cells analyzed 15 h post-transfection of the plasmids as indicated; green color shows an expression of GFP, p60AmotL2DILI (containing a three a.a. deletion in the C-terminus) and p60AmotL2-GFP, respectively. Actin is visualized by phalloidin (red) and nuclei by DAPI (blue) staining. Images were captured at a magnification of 63×. Scale bar = 5 µm. (**B**) Excerpts from live imaging (Supplemental Movie S1) 8h post-transfection of MDCK cells stably expressing E-cadherin-RFP and transfected with a p60AmotL2-GFP plasmid. Control excerpts and movies are shown in supplemental data. Arrows indicate positive cells. Magnification 40×. (**C**) Bar diagram shows the quantification of GFP-positive extruding cells at fixed and stained 15 h post-transfection (Mann–Whitney U-test, *** *p* < 0.001). (**D**) Bar diagram shows the quantification of GFP-positive extruding cells as analyzed by live imaging accumulated from 8 to 24h post-transfection (Mann–Whitney U-test, *** *p* < 0.001). (**E**) The graph depicts the quantification of extruded GFP-positive cells over time using the Incucyte live cell analysis system. The data represents the mean values of three replicate wells from three independent experiments per time point, shown as the mean ± SD (*t*-test). Significant differences in cell number were observed at every time point from time points 16 h to 40 h.

To establish a causal relationship between p60AmotL2 expression and apical cell extrusion, MDCK cells stably expressing E-cadherin-tagged with a red fluorescent protein (MDCK-E-cad-RFP) were transfected with a p60AmotL2 plasmid tagged with a green fluorescent protein (p60AmotL2-GFP). Control cells were transfected with either a myristylated-GFP construct or a p60Amotl2 construct featuring a mutated PDZ binding domain (p60AmotL2ΔILI-GFP), which impacts AmotL2 protein localization and activity [29]. Cells were fixed 15h post-transfection and analyzed by confocal microscopy. The 3D rendering in the XZ axis showed the rosette formation and rounding up of extruding p60AmotL2-expressing cells. In contrast, the control cells and p60AmotL2ΔILI-GFP cells showed a different behavior as they remained embedded within the epithelial layer. (Figure 4A and quantified in bar diagram in Figure 4C). Time-lapse imaging over 15 h revealed that p60AmotL2-GFP formed vesicles initially localizing to cell-cell junctions before internalizing (Supplemental Movie S1). This internalization coincided with the formation of rosette-like structures by neighboring cells, eventually leading to the extrusion of the p60AmotL2-positive cell (Figure 4B and quantified in bar diagram in 4D). Apical extrusion was further analyzed using the IncuCyte Live-Cell Analysis System, which allows for the automatic acquisition and analysis of cell images with minimal perturbation to cell viability. Using this system, we identified and quantified cell extrusion events in a non-biased manner, as shown in Supplemental Movies S2 and S3. Image analysis demonstrated a significant induction of apical extrusion in p60AmotL2-expressing cells (Figure 4E).

### 3.5. p60AmotL2-Induced Apical Extrusion Is INDEPENDENT of Apoptosis

**Figure 5:**
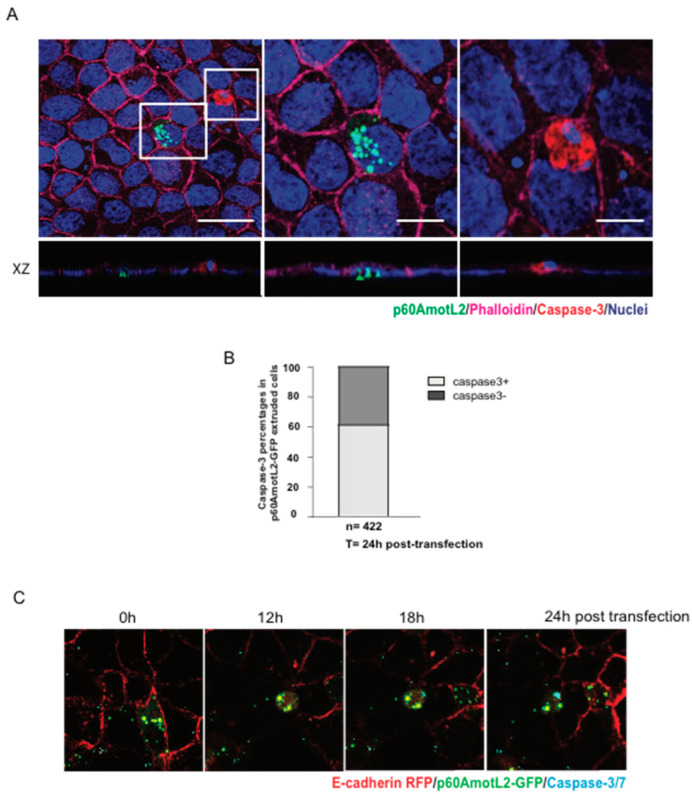
p60AmotL2 expression does not negatively impact cell viability. (**A**) A representative immunofluorescence image of activated Caspase-3 in MDCK control cells after transfection with the p60AmotL2-GFP plasmid. The white square to the left highlights p60AmotL2-GFP positive cells with a rosette formation formed by neighboring cells. The other square shows a GFP-negative apoptotic cell positive for active Caspase-3. Scale bar left panel = 20 µm, middle and right panel = 10 µm. (**B**) Quantification of the percentage of cells that are positive for p60AmotL2-GFP and Caspase-3 24 h post-transfection (*n* = 422, Mann–Whitney U-test). (**C**) Excerpts from a time-lapse movie of 40 h started at 8 h post-transfection (Supplementary Movie S4) show that p60AmotL2-GFP induces extrusion, which precedes Caspase-3/7 positivity. Data are presented as mean ± SD from three independent experiments.

Considering that apoptotic cells may initiate signals leading to apical extrusion, we investigated whether p60AmotL2-triggered apoptosis could explain our observations. We assessed the effect of p60AmotL2 expression on cell viability using several approaches. First, a clonogenicity assay measuring the ability of single cells to proliferate and form colonies showed no negative impact of p60AmotL2 expression on colony formation (Supplementary Figure S1A,B). Secondly, the majority of extruding p60AmotL2-positive cells did not exhibit caspase-3 activation (Figure 5A,B). Additionally, live imaging demonstrated that p60AmotL2 expression and apical extrusion preceded caspase-3 activation (Figure 5C and Supplementary Movie S4). Collectively, our findings suggest that p60AmotL2 does not activate a pro-apoptotic program or adversely affect cell viability, strengthening the causal link between p60AmotL2 expression and apical extrusion.

### 3.6. p60AmotL2 Promotes Cell Growth beyond Typical Contact Inhibition Limits

**Figure 6:**
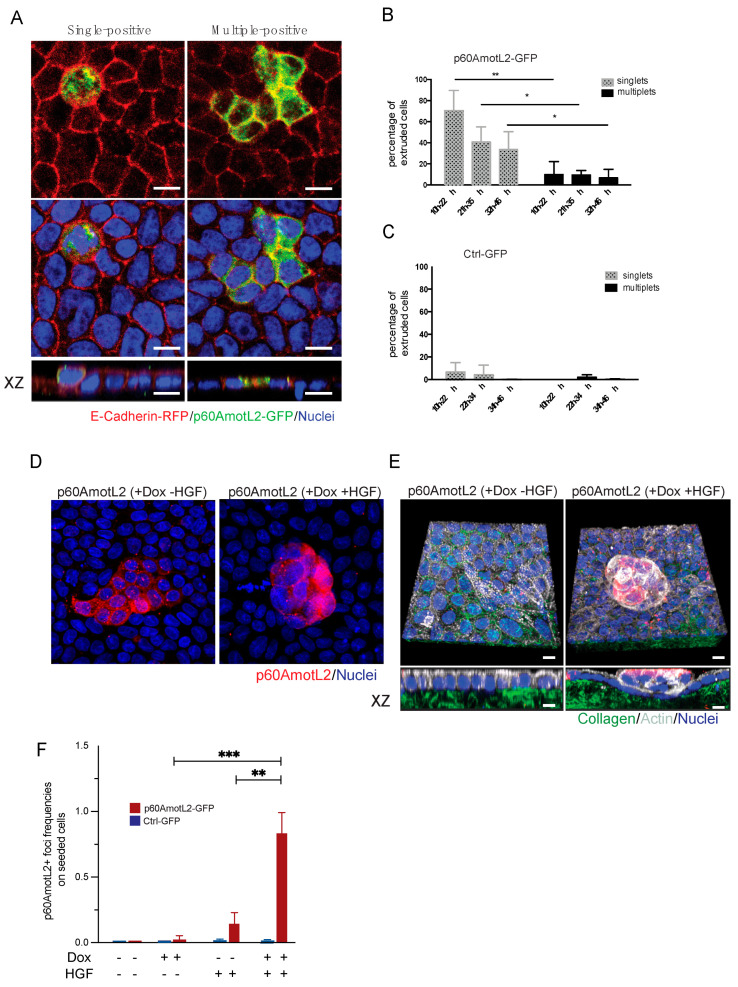
p60AmotL2 expression bypasses contact inhibition. (**A**) Immunofluorescence imaging displays the extrusion of a single p60AmotL2-GFP positive cell (left panel), while clusters of positive cells remain embedded in the epithelial layer (right panel). (**B**,**C**) Bar diagrams illustrate the quantification of extrusion percentages of single or multiple p60AmotL2-GFP or GFP-control cells (Mann–Whitney U-test, ** *p* < 0.01, * *p* < 0.05). (**D**) Dox-regulated p60AmotL2 cells were plated at a 1:50 ratio with wild-type MDCK cells on Collagen 1 matrix. Stimulation with HGF induced loss of contact inhibition and focus formation. (**E**) 3-D rendering of cells shown in D. Collagen was labeled with Oregon Green and actin visualized with phalloidin (shown in white) (**F**) Graph displaying the number of p60-positive foci formed after Dox and HGF stimulation as indicated (Mann–Whitney U-test, *** *p* < 0.001, ** *p* < 0.01). Scale bar = 10 µm.

The extrusion process relies on the contractile properties of neighboring cells, which form a rosette and expel unwanted cells through cell-cell interactions and actomyosin contractility. In our experiments, we observed that single p60AmotL2-expressing cells were extruded, while clusters of three or more expressing cells remained embedded within the epithelial layer (Figure 6A,B). Our previous work demonstrated that p60AmotL2 inhibits radial actin filament formation and E-cadherin force transmission. We sought to determine whether this inhibition could provide a growth advantage and a mechanism for overcoming contact inhibition. When hepatocyte growth factor (HGF) was added to confluent MDCK cells, it did not activate cell proliferation (Figure 6D). Subsequently, we mixed doxycycline-regulated p60AmotL2-transfected cells with wild-type MDCK cells at a 1:50 ratio and plated them to reach 100% confluency. Induction of p60AmotL2 resulted either in extrusion or clusters of induced cells that were still integrated with the epithelial layer (as shown in Figure 6A). However, p60AmotL2 expression combined with HGF stimulation, led to a loss of contact inhibition, allowing cells to outgrow the epithelial layer and form spheroid-like colonies (Figure 6 D,E). These results suggest that p60AmotL2 confers a growth advantage and a mechanism to bypass contact inhibition in MDCK cells.

### 3.7. BioID Analysis of the AmotL2 Adhesome

**Figure 7:**
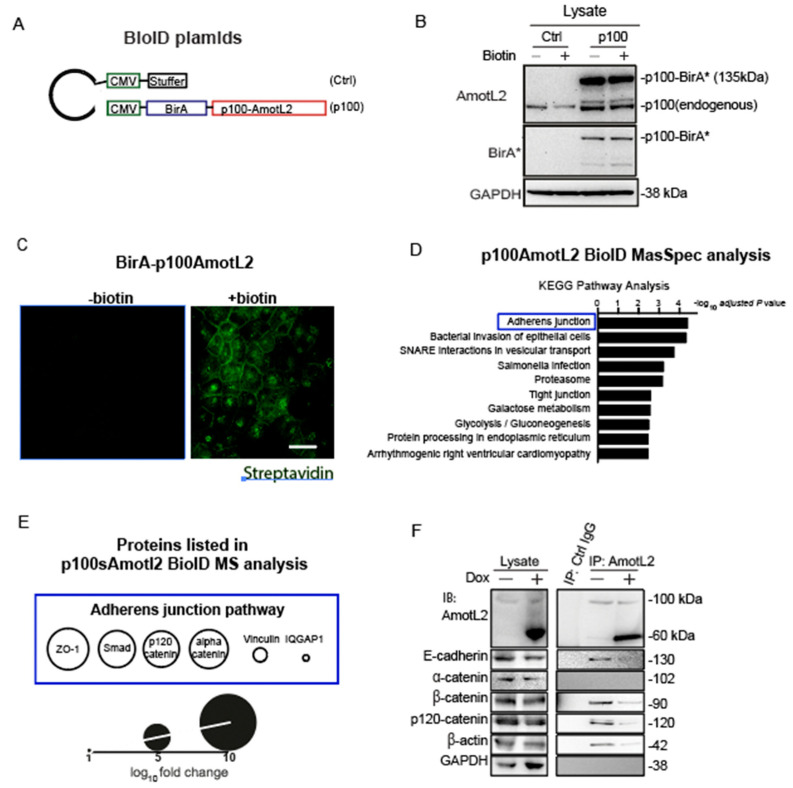
p60AmotL2 interferes with E-cadherin/p120 catenin interaction. (**A**) Schematic representation of the plasmid constructs used for BioID. (**B**) Western blot analysis of the expression of BirA-p100AmotL2 in MDCK cells. BirA* stands for the R118G mutated BirA(reference). (**C**) Immunofluorescence streptavidin staining shows that BirA-p100AmotL2 hybrid proteins localize to the cellular junctions. Scale bar = 20 µm. (**D**) KEGG pathway analysis of p100AmotL2 binding proteins. (**E**) Proteins in the adherens junction pathway that are in close proximity to p100AmotL2. (**F**) Immunoprecipitation analysis of AmotL2 in the absence or presence of p60AmotL2. Note that p60AmotL2 induction decreases the binding of E-cadherin, β-actin and p120catenin.

To elucidate the molecular mechanisms by which p60AmotL2 promotes apical extrusion, we sought to identify proteins that bind directly or are in close proximity to AmotL2. For this purpose, we employed the proximity-dependent biotin identification (BioID) method, which involves fusing the promiscuous biotin ligase BirA to the protein of interest. Upon expression in cells, BirA can biotinylate interacting proteins [30]. We fused BirA to the N-terminus p100 isoform (depicted schematically in Figure 7A). The constructs were transfected into MDCK cells and verified using antibodies against BirA and LDS-AmotL2 (Figure 7B). After adding biotin for a duration of 16 h, protein biotinylation occurred within a proximity of 10 nm by BirA-tagged p100AmotL2, which was localized to the cell-cell junction in confluent cells. The biotinylated proteins were effectively pulled down using streptavidin beads, as confirmed by Western Blot analysis (Supplementary Figure S2A,B).

The biotinylated interactors of p100AmotL2 were identified through mass spectrometry. 186 proteins, exhibiting a fold change (FC) greater than 0, were considered positive hits and documented in Table S1. Further analysis of these positive hits using KEGG pathway analysis (Enrichr) revealed that the ‘Adherens Junctions’ pathway exhibited the highest enrichment in association with p100AmotL2 (Figure 7D, Table S2). Junctional proteins identified by mass spectrometry included Zona Occludens-1 (ZO1), Mothers against decapentaplegic homolog 3 (SMAD3), p120-catenin, alpha-catenin, vinculin, and Ras GTPase-activating-like protein 1 (IQGAP1) (Figure 7E). We focused on alpha- and p120-catenin, as they likely mediated the binding of AmotL2 to E-cadherin. Only the association of p120 catenin with p100AmotL2 was confirmed by immunoprecipitation analysis (Figure 7F). Expression of p60AmotL2 inhibited the association with E-cadherin, p120 catenin and actin (Figure 7F). In conclusion, our results indicate that p100 AmotL2 is associated with E-cadherin via binding to p120 catenin and that p60Amotl2 dissociates this interaction.

## 4. Discussion

**Figure 8:**
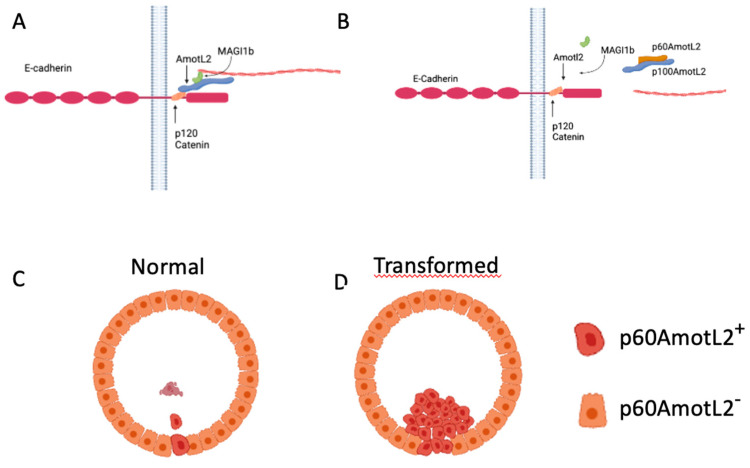
Hypothetical Model of p60AmotL2 in Normal and Pathological Extrusion. (**A**) AmotL2 p100 Interaction with E-Cadherin and Actin Filament Formation: AmotL2 p100 associates with E-cadherin through interaction with p120 Catenin, triggering actin filament formation. (**B**) Dominant-Negative Effect of p60AmotL2: p60AmotL2 functions as a dominant-negative factor, inhibiting the connection between p100AmotL2 and the E-cadherin junctional complex, as well as impeding actin filament formation. (**C**) Single Cell Expression of p60AmotL2: Cells expressing p60AmotL2 undergo extrusion and subsequently undergo anoikis. (**D**) Immortalized Cells Expressing p60AmotL2: Immortalized cells expressing p60AmotL2 evade anoikis, leading to sustained proliferation within the glandular lumen.

Here we report for the first time that p60AmotL2 is an inducer of apical extrusion. Our findings are in line with data showing that actomyosin contractility plays an important role in apical extrusion. E-cadherin organizes epithelial cells by coupling cells to contractile actin cytoskeleton [31]. E-cadherin not only acts as a junctional complex connecting cells but is also of importance for relaying mechanical forces between neighboring cells and preventing the de-lamination of individual cells [32]. The precise control of tensile activity appears to be critical not only for the function of the epithelium but may also determine which cells are destined for extrusion. For example, the Rho-Kinase (ROCK) is required for spontaneous as well as oncogenic-driven extrusion of cells [12]. Similar findings have been shown with the small GTPase CDC42, which is involved in the organization of the cytoskeleton [33]. We have previously shown that E-cadherin is dependent on p100AmotL2 to generate radial actin filaments that relay force between cells [26]. Furthermore, the p60AmotL2 isoform acts as an antagonist to the p100AmotL2/E-cadherin complex, disconnecting radial actin and thereby decreasing tensile force (Schematic in Figure 8). Here we further report that p100AmotL2 is associated with p120catenin and that this complex is inhibited by the expression of p60AmotL2. This is of interest as p120catenin has previously been shown to play a role in cell extrusion. Research by Kourtidis et al. has illustrated that apical and basal E-cadherin-based junctions have contrasting roles in growth regulation, contingent on the activity of p120catenin and Src, where apical complexes inhibit the growth and EMT-promoting effects of Src and p120catenin via the microRNA miR-30b [34].

The p60AmotL2 is normally expressed at low or non-detectable levels in culture. Specific stress signals such as hypoxia activate transcription of p60AmotL2 by the binding of c-Fos to its promoter. How p60AmotL2 is activated on a single cell level remains to be elucidated. It does, however, indicate that the initial signal to be extruded comes from the targeted cell. We hypothesize that the decrease in radial actin and a concomitant decrease in tensile strength triggers the formation of a contractile actin ring in the neighboring cells that ultimately squeezes out the redundant cell. However, as we have previously shown that p60AmotL2 is a negative regulator of apical-basal polarity we cannot exclude that this may also affect cell extrusion.

As p60AmotL2 appears to be expressed in extruding cells it is possible that p60AmotL2 expression is pro-apoptotic and thereby triggers extrusion. However, data shown here indicates that cells being extruded by this mechanism are initially live as they have intact membranes, and markers of Caspase-3 activity were not present. Furthermore, p60AmotL2-expressing cells retained an intact capacity to grow in a colony formation assay, indicating that p60AmotL2 expression is not deleterious to the cells. Additionally, it was shown that clusters of p60AmotL2 positive cells were maintained in the epithelial layer without being extruded or undergoing cell death. This is similar to results indicated in glandular cancer such as the prostate where the high intensity of vesicular AmotL2 could be detected without apparent shedding into the lumen. Collectively this indicates that singular cells expressing p60AmotL2 are prone to extrusion, whereas groups of cells remain within the epithelial monolayer (Figure 8).

This investigation has centered on elucidating the functional role of p60AmotL2 in the apical extrusion mechanism. However, it is noteworthy that in vitro activation of oncogenes results in an opposing basal extrusion phenomenon. Marshall and colleagues demonstrated a transition from apical to basal extrusion through the inactivation of the Adenomatosis Polyposis Coli (APC) tumor suppressor gene [35]. The implications of this directional shift on the viability of shed tumor cells remain enigmatic. In scenarios such as ductal carcinoma in situ (DCIS) of the breast, where tumor cells colonize ducts, one might speculate on an apical mode of extrusion. As the evidence accumulates, the induction of apical extrusion by p60AmotL2 further supports the notion that tumor cells co-opt ordinary cellular processes to fortify their survival and proliferation [17]. Subsequent investigations will whether p60AmotL2-expressing cells exhibit distinct susceptibility to cancer therapies. In conclusion, our findings provide insights into the intricate interplay between normal and malignant cell dynamics, shedding light on potential avenues for therapeutic intervention.

## 5. Conclusions

Our study reveals a physiological function of p60AmotL2 in regulating homeostasis through the activation of apical extrusion. The impact of this isoform on E-cadherin and its connection to invasive tumors provides insights into a potential mechanism for tu-mor cell escape. Through our investigation of extrusion dynamics and p60AmotL2′s expression, we uncover a potential pathway through which tumor cells harness this process for invasion.

## Data Availability

No new data were created or analyzed in this study. Data sharing is not applicable to this article.

## Supplementary Materials

The following supporting information can be downloaded at: https://www.mdpi.com/article/10.3390/cells12172158/s1, Supplemental Figure S1: (A) Representative live cell imaging excerpts of MDCK cells expressing E-cadherin-RFP and transfected with p60AmotL2ΔILI-GFP or Control-GFP plasmids. Representative images from live imaging movies are shown, captured over a 15-h period post-transfection of MDCK cells. The cells expressed E-cadherin tagged with red fluorescent protein (E-cadherin-RFP) and were transfected with either p60AmotL2ΔILI-GFP or Control-GFP plasmids. (B) Impact of p60AmotL2 expression on clonogenicity assessed by a colony formation assay. MDCK cells were transfected with either p60AmotL2 or control plasmids, and their clonogenicity was assessed using a colony formation assay. The experiment was performed in triplicates, with 1000 cells plated in 10 cm petri dishes. The cells were allowed to form colonies over a period of approximately 7 days. Following the incubation period, the colonies were fixed with 4% PFA and stained with crystal violet to visualize and distinguish individual colonies. The number of colonies was quantified using either image analysis software or manual counting methods. The data presented in Figure B represent the mean ± SD derived from three independent experiments. Statistical analysis (Mann–Whitney U-test) revealed no significant difference in clonogenicity between cells expressing p60AmotL2 and control cells. These results indicate that the expression of p60AmotL2 does not impact clonogenicity in MDCK cells. (D) Representative figures of the GFP-ctrl groups, as depicted in Figure 6A. Supplemental Figure S2: (A) Western blot analysis was performed to examine the expression of BioID-fusion protein in transfected MDCK cells. The analysis included samples from cells transfected with an empty vector and p100AmotL2-BioID construct. To induce biotinylation, biotin was added to the cells for 16 h. (B) Immunofluorescence staining was conducted on transfected MDCK cells to visualize streptavidin (green), BirA (red), and nuclei (DAPI). (C) Immunofluorescence staining was performed on wild-type and Dox-regulated p60AmotL2 MDCK cells to investigate the presence and localization of AmotL2 (green), p120catenin (red), and nuclei (DAPI). The staining enabled us to observe the distribution of these proteins in the different cell types. In all images, a scale bar of 20 µm was used to provide a reference for the size of the observed structures. Supplemental Figure S3: (A) Full blot for Figure 2B. (B) Full blot for Figure 3B. Supplemental Movie S1: Representative movie of p60AmotL2-GFP cells extrusion, related to Figure 4B. Scale bar = 20 µm. Supplemental Movie S2: Representative Incucyte-captured image sequences of MDCK cells transfected with GFP-Ctrl plasmids over 46 h, related to Figure 4E. Scale bar = 200 µm. Supplemental Movie S2: Representative Incucyte-captured image sequences of MDCK cells transfected with GFP-Ctrl plasmids over 46 h, related to Figure 4E. Scale bar = 200 µm. Supplemental Movie S3: Representative Incucyte-captured image sequences of MDCK cells transfected with GFP-Ctrl plasmids over 46 h, related to Figure 4E. Scale bar = 200 µm. Supplemental Movie S4: Representative movie related to Figure 5C, showing p60AmotL2 expression and apical extrusion preceded caspase-3 activation. Size and time scales are embedded into the movie. Scale bar = 20 µm. Supplemental Table S1: List of AmotL2 binding proteins identified through mass spectrometry. Supplemental Table S2: KEGG pathway analysis of proteins identified by AmotL2 BioID data.
