# Supplementary material for: Deciphering the Role of p60AmotL2 in Epithelial Extrusion and Cell Detachment
Source: Cells. 2023 Aug 28;12(17):2158. doi: 10.3390/cells12172158 (PMC10486482; doi:10.3390/cells12172158)
Supplement: Supplementary file 1 [file cells-12-02158-s001.zip › Table S1 MDCK Amotl2 BioID list.pdf]

empty vector -biotin			empty vector +biotin			p100 -biotin			p100 +biotin			
0,000E0	0,000E0	0,000E0	0,000E0	0,000E0	0,000E0	0,000E0	0,000E0	0,000E0	1,070E8	1,538E8	1,480E8	AMOTL2
0,000E0	0,000E0	0,000E0	0,000E0	0,000E0	0,000E0	0,000E0	0,000E0	0,000E0	1,858E8	2,320E8	4,693E7	AHNAK
8,142E7	3,170E5	1,105E7	2,094E8	1,004E8	1,356E8	8,664E6	4,568E8	2,880E8	1,107E9	1,496E9	7,230E8	PC
4,036E6	0,000E0	1,053E6	4,076E7	1,847E7	2,068E7	1,860E6	4,680E7	4,013E7	3,021E8	4,695E8	2,114E8	MCCC1
0,000E0	0,000E0	2,430E5	1,189E6	3,580E5	4,716E5	0,000E0	6,020E6	8,710E6	1,413E7	2,196E7	1,741E7	RAB1A
2,012E8	2,976E6	1,508E7	1,319E8	5,619E7	8,435E7	1,778E7	6,187E8	5,268E8	7,998E8	1,238E9	6,593E8	ACTC1
1,128E7	0,000E0	2,430E5	1,339E6	1,483E6	1,140E6	0,000E0	7,024E6	1,065E7	1,674E7	2,251E7	1,639E7	RAB1B
2,429E6	0,000E0	0,000E0	1,948E6	3,038E6	3,769E5	7,274E4	8,260E6	8,037E6	5,000E7	2,414E7	1,478E7	-//-
0,000E0	0,000E0	0,000E0	0,000E0	0,000E0	0,000E0	0,000E0	0,000E0	0,000E0	5,215E7	6,955E7	5,673E7	KIAA1217
2,071E7	0,000E0	6,591E5	6,589E6	4,107E6	5,281E6	0,000E0	4,397E7	2,168E7	6,569E7	8,777E7	5,067E7	MYH9
0,000E0	0,000E0	0,000E0	0,000E0	0,000E0	0,000E0	0,000E0	0,000E0	0,000E0	2,077E6	5,670E6	5,932E6	YAP1
0,000E0	0,000E0	0,000E0	0,000E0	0,000E0	0,000E0	0,000E0	0,000E0	0,000E0	2,866E6	2,536E7	8,666E6	CTTN
2,158E6	0,000E0	0,000E0	2,254E5	1,881E6	0,000E0	0,000E0	2,262E6	8,397E6	8,844E6	2,081E7	2,221E7	NQO1
2,799E6	0,000E0	0,000E0	5,839E5	0,000E0	0,000E0	0,000E0	6,192E6	7,618E6	1,483E7	1,438E7	9,178E6	EPCAM
9,478E5	0,000E0	6,662E5	0,000E0	7,610E5	1,366E6	0,000E0	5,741E6	5,861E6	6,881E6	1,293E7	9,818E6	CCT8
0,000E0	0,000E0	0,000E0	0,000E0	0,000E0	0,000E0	0,000E0	0,000E0	0,000E0	2,264E6	5,971E6	1,717E6	CRK
0,000E0	0,000E0	0,000E0	0,000E0	0,000E0	0,000E0	0,000E0	0,000E0	0,000E0	0,000E0	6,116E5	1,966E6	SEC61B
1,064E7	0,000E0	0,000E0	0,000E0	1,888E6	0,000E0	0,000E0	0,000E0	0,000E0	3,187E7	4,323E7	2,503E7	RPL30
3,138E6	0,000E0	0,000E0	3,434E5	0,000E0	0,000E0	0,000E0	1,386E6	3,449E6	4,475E6	7,703E6	3,863E6	G6PD
0,000E0	0,000E0	0,000E0	0,000E0	0,000E0	0,000E0	0,000E0	0,000E0	0,000E0	7,404E6	2,195E7	1,143E7	LOC102155463
1,091E6	0,000E0	0,000E0	0,000E0	0,000E0	0,000E0	0,000E0	0,000E0	0,000E0	2,419E6	3,236E6	5,150E6	LAD1
3,161E6	0,000E0	2,992E5	7,168E5	3,773E5	1,119E6	0,000E0	7,994E6	1,929E6	1,421E7	2,856E7	1,080E7	FLNA
0,000E0	0,000E0	0,000E0	0,000E0	0,000E0	0,000E0	0,000E0	0,000E0	0,000E0	0,000E0	9,213E5	7,831E5	GSTK1
0,000E0	0,000E0	0,000E0	0,000E0	0,000E0	0,000E0	0,000E0	0,000E0	0,000E0	2,802E7	4,907E7	1,613E7	EIF4ENIF1
0,000E0	0,000E0	0,000E0	0,000E0	0,000E0	0,000E0	0,000E0	0,000E0	0,000E0	2,103E7	3,516E7	2,682E7	SEC16A
0,000E0	0,000E0	0,000E0	0,000E0	0,000E0	0,000E0	0,000E0	0,000E0	0,000E0	1,467E7	2,333E6	2,422E6	CORO1B
6,796E5	0,000E0	0,000E0	7,913E5	1,932E5	0,000E0	0,000E0	3,350E6	1,200E6	6,812E6	1,683E7	5,610E6	JUP
0,000E0	0,000E0	0,000E0	0,000E0	0,000E0	0,000E0	0,000E0	0,000E0	0,000E0	5,347E5	2,836E6	2,541E6	STX7
0,000E0	0,000E0	0,000E0	0,000E0	0,000E0	0,000E0	0,000E0	0,000E0	0,000E0	1,438E6	1,487E6	3,055E6	LOC403934
0,000E0	0,000E0	0,000E0	0,000E0	4,411E5	0,000E0	0,000E0	1,900E6	1,693E6	3,789E6	9,998E6	5,405E6	ASNS
0,000E0	0,000E0	0,000E0	3,372E5	0,000E0	0,000E0	0,000E0	0,000E0	0,000E0	1,197E6	8,223E6	3,951E6	ACAD9
0,000E0	0,000E0	0,000E0	0,000E0	0,000E0	0,000E0	0,000E0	0,000E0	0,000E0	9,981E5	5,911E6	0,000E0	SEC22B
0,000E0	0,000E0	0,000E0	0,000E0	0,000E0	0,000E0	0,000E0	0,000E0	0,000E0	0,000E0	6,793E5	7,676E5	ESD
0,000E0	0,000E0	0,000E0	0,000E0	0,000E0	0,000E0	0,000E0	0,000E0	0,000E0	9,746E6	1,658E7	1,134E7	RANBP2
0,000E0	0,000E0	0,000E0	0,000E0	0,000E0	0,000E0	0,000E0	0,000E0	0,000E0	1,409E6	0,000E0	1,771E6	VAMP3
0,000E0	0,000E0	0,000E0	0,000E0	0,000E0	0,000E0	0,000E0	0,000E0	0,000E0	1,431E6	4,396E6	2,518E6	CTNNA1
0,000E0	0,000E0	0,000E0	0,000E0	0,000E0	0,000E0	0,000E0	0,000E0	0,000E0	9,109E5	4,149E6	3,269E5	NDUFC2
1,291E6	0,000E0	0,000E0	1,501E5	0,000E0	0,000E0	0,000E0	3,496E6	2,541E6	7,328E6	4,483E6	7,540E6	FLNB
1,396E6	0,000E0	0,000E0	0,000E0	0,000E0	0,000E0	0,000E0	0,000E0	0,000E0	9,254E6	4,662E6	1,700E6	SEC14L2
0,000E0	0,000E0	0,000E0	0,000E0	0,000E0	0,000E0	0,000E0	0,000E0	0,000E0	2,998E6	1,527E6	1,678E6	CKAP4
0,000E0	0,000E0	0,000E0	0,000E0	0,000E0	0,000E0	0,000E0	0,000E0	0,000E0	0,000E0	1,223E6	2,902E5	DAD1
3,880E6	0,000E0	0,000E0	0,000E0	5,275E5	0,000E0	0,000E0	0,000E0	4,313E6	7,478E6	7,080E6	5,373E6	SHMT2
0,000E0	0,000E0	0,000E0	0,000E0	0,000E0	0,000E0	0,000E0	0,000E0	0,000E0	5,812E5	2,018E6	6,210E5	IGF2BP1
5,382E6	0,000E0	0,000E0	2,282E6	3,062E6	0,000E0	0,000E0	5,117E6	6,663E6	1,527E7	2,060E7	1,319E7	LRPPRC
0,000E0	0,000E0	0,000E0	0,000E0	0,000E0	0,000E0	0,000E0	0,000E0	8,367E5	3,973E6	5,911E6	9,211E5	-//-
0,000E0	0,000E0	0,000E0	0,000E0	0,000E0	0,000E0	0,000E0	4,038E6	2,966E6	1,640E7	1,222E7	5,262E6	GPI
1,740E6	0,000E0	0,000E0	1,947E5	4,101E5	0,000E0	0,000E0	2,574E6	1,376E6	4,391E6	9,890E6	8,158E6	GLS
5,679E5	0,000E0	0,000E0	3,431E5	0,000E0	0,000E0	0,000E0	0,000E0	0,000E0	3,146E6	2,066E6	2,347E6	RUVBL1
0,000E0	0,000E0	0,000E0	0,000E0	0,000E0	0,000E0	0,000E0	0,000E0	0,000E0	4,111E6	9,173E6	1,091E7	TJP1
0,000E0	0,000E0	0,000E0	0,000E0	0,000E0	0,000E0	0,000E0	0,000E0	1,118E5	1,322E6	6,870E6	3,266E6	CDK1
0,000E0	0,000E0	0,000E0	0,000E0	0,000E0	0,000E0	0,000E0	0,000E0	0,000E0	0,000E0	4,645E7	1,300E6	-//-
0,000E0	0,000E0	0,000E0	0,000E0	0,000E0	0,000E0	0,000E0	0,000E0	0,000E0	1,494E6	0,000E0	6,354E5	VAMP5
0,000E0	0,000E0	0,000E0	0,000E0	0,000E0	0,000E0	0,000E0	3,248E6	0,000E0	5,508E6	8,770E6	5,020E6	RPN1
0,000E0	0,000E0	0,000E0	0,000E0	0,000E0	0,000E0	0,000E0	0,000E0	0,000E0	9,603E5	1,582E6	1,403E6	EPS8L2
0,000E0	0,000E0	0,000E0	0,000E0	1,232E5	0,000E0	0,000E0	0,000E0	0,000E0	1,950E6	5,834E6	7,718E5	VCL

0,000E0	0,000E0	0,000E0	0,000E0	0,000E0	0,000E0	0,000E0	0,000E0	0,000E0	1,808E6	2,407E6	2,561E6	PKP4
0,000E0	0,000E0	0,000E0	0,000E0	0,000E0	0,000E0	0,000E0	3,998E5	0,000E0	9,843E5	8,538E5	6,000E5	ARPC2
0,000E0	0,000E0	0,000E0	0,000E0	0,000E0	0,000E0	0,000E0	0,000E0	0,000E0	3,767E5	1,302E6	7,393E5	RAB11A
0,000E0	0,000E0	0,000E0	0,000E0	0,000E0	0,000E0	0,000E0	0,000E0	0,000E0	0,000E0	1,052E6	3,044E5	DPYSL2
0,000E0	0,000E0	0,000E0	0,000E0	0,000E0	0,000E0	0,000E0	3,529E6	2,978E6	8,039E6	5,085E6	6,009E6	LIMA1
0,000E0	0,000E0	0,000E0	0,000E0	0,000E0	0,000E0	0,000E0	0,000E0	0,000E0	0,000E0	1,665E6	2,559E6	PSME3
1,908E6	0,000E0	0,000E0	0,000E0	0,000E0	0,000E0	0,000E0	7,580E5	1,066E6	2,983E6	5,410E6	4,684E6	PFKP
1,680E6	0,000E0	0,000E0	1,013E6	3,807E5	0,000E0	0,000E0	4,514E6	2,836E6	8,263E6	1,799E7	5,783E6	CLTC
0,000E0	0,000E0	0,000E0	0,000E0	0,000E0	0,000E0	0,000E0	0,000E0	0,000E0	5,286E5	1,226E6	7,325E5	PDCD6
0,000E0	0,000E0	0,000E0	0,000E0	0,000E0	0,000E0	0,000E0	0,000E0	1,854E6	5,188E6	6,112E6	2,631E6	BZW2
6,812E5	0,000E0	0,000E0	1,072E5	0,000E0	0,000E0	0,000E0	0,000E0	1,455E6	3,788E6	1,069E7	4,016E6	PCK2
4,391E5	0,000E0	0,000E0	0,000E0	0,000E0	0,000E0	0,000E0	0,000E0	0,000E0	9,204E5	2,407E6	7,868E5	CNPY2
0,000E0	0,000E0	0,000E0	0,000E0	0,000E0	0,000E0	0,000E0	0,000E0	0,000E0	0,000E0	3,352E6	2,733E6	-//-
2,201E6	0,000E0	0,000E0	0,000E0	0,000E0	0,000E0	0,000E0	0,000E0	0,000E0	5,885E6	2,351E6	1,054E7	PRDX6
0,000E0	0,000E0	0,000E0	1,517E5	1,542E5	0,000E0	0,000E0	0,000E0	0,000E0	3,187E5	6,800E5	5,074E5	-//-
0,000E0	0,000E0	0,000E0	0,000E0	0,000E0	0,000E0	0,000E0	0,000E0	0,000E0	1,306E6	1,558E6	9,333E5	TBRG4
0,000E0	0,000E0	0,000E0	0,000E0	0,000E0	0,000E0	0,000E0	0,000E0	0,000E0	0,000E0	9,427E6	8,827E5	PTCD3
0,000E0	0,000E0	0,000E0	0,000E0	0,000E0	0,000E0	0,000E0	0,000E0	0,000E0	1,308E6	4,300E6	0,000E0	WARS1
0,000E0	0,000E0	0,000E0	7,915E5	0,000E0	0,000E0	0,000E0	0,000E0	1,861E6	1,108E7	1,789E7	4,647E6	CANX
0,000E0	0,000E0	0,000E0	0,000E0	0,000E0	0,000E0	0,000E0	0,000E0	0,000E0	3,591E6	9,867E6	4,563E6	SSR4
0,000E0	0,000E0	0,000E0	0,000E0	0,000E0	0,000E0	0,000E0	0,000E0	0,000E0	0,000E0	5,605E5	3,372E6	VCPIP1
0,000E0	0,000E0	0,000E0	0,000E0	0,000E0	0,000E0	0,000E0	0,000E0	0,000E0	2,195E6	4,936E6	1,950E6	ERC1
0,000E0	0,000E0	0,000E0	0,000E0	0,000E0	0,000E0	0,000E0	0,000E0	0,000E0	6,846E5	6,918E5	0,000E0	MAVS
0,000E0	0,000E0	0,000E0	0,000E0	0,000E0	0,000E0	0,000E0	0,000E0	0,000E0	7,072E5	1,242E6	1,489E6	YWHAG
0,000E0	0,000E0	0,000E0	0,000E0	0,000E0	0,000E0	0,000E0	0,000E0	0,000E0	4,848E5	5,961E5	0,000E0	CRIP2
0,000E0	0,000E0	0,000E0	0,000E0	0,000E0	0,000E0	0,000E0	0,000E0	0,000E0	1,632E6	6,221E5	7,353E5	SRP68
0,000E0	0,000E0	0,000E0	0,000E0	0,000E0	0,000E0	0,000E0	0,000E0	0,000E0	0,000E0	1,591E6	6,751E5	GNA11
0,000E0	0,000E0	0,000E0	0,000E0	0,000E0	0,000E0	0,000E0	0,000E0	1,895E6	3,971E6	3,588E6	2,134E6	PRXL2A
0,000E0	0,000E0	0,000E0	0,000E0	0,000E0	0,000E0	0,000E0	2,363E6	0,000E0	1,325E7	1,347E7	1,085E7	RBM47
0,000E0	0,000E0	0,000E0	0,000E0	0,000E0	0,000E0	0,000E0	0,000E0	0,000E0	1,084E6	8,571E6	7,329E5	EIF4G1
0,000E0	0,000E0	0,000E0	0,000E0	0,000E0	0,000E0	0,000E0	0,000E0	0,000E0	0,000E0	2,931E6	7,705E5	LBR
0,000E0	0,000E0	0,000E0	0,000E0	0,000E0	0,000E0	0,000E0	0,000E0	1,118E5	1,859E6	6,870E6	5,872E6	CDK6
0,000E0	0,000E0	0,000E0	0,000E0	0,000E0	0,000E0	0,000E0	0,000E0	0,000E0	4,875E6	4,172E6	6,613E6	PATJ
0,000E0	0,000E0	0,000E0	0,000E0	0,000E0	0,000E0	0,000E0	0,000E0	0,000E0	6,206E6	7,655E6	1,334E7	PKP2
0,000E0	0,000E0	0,000E0	0,000E0	0,000E0	0,000E0	0,000E0	0,000E0	0,000E0	8,599E5	1,634E6	1,437E6	KARS1
0,000E0	0,000E0	0,000E0	0,000E0	0,000E0	0,000E0	0,000E0	0,000E0	0,000E0	9,946E5	5,183E6	0,000E0	LSM2
0,000E0	0,000E0	0,000E0	0,000E0	0,000E0	0,000E0	0,000E0	0,000E0	0,000E0	0,000E0	4,318E6	4,201E6	MACC1
0,000E0	0,000E0	0,000E0	0,000E0	0,000E0	9,459E5	0,000E0	0,000E0	0,000E0	8,071E6	7,909E6	2,061E6	VAT1
0,000E0	0,000E0	0,000E0	0,000E0	0,000E0	0,000E0	0,000E0	0,000E0	0,000E0	2,820E6	4,434E6	4,764E6	ARHGAP21
0,000E0	0,000E0	0,000E0	0,000E0	0,000E0	0,000E0	0,000E0	0,000E0	0,000E0	4,885E5	0,000E0	1,337E6	BSG
0,000E0	0,000E0	0,000E0	0,000E0	0,000E0	0,000E0	0,000E0	0,000E0	0,000E0	2,793E5	1,298E6	7,329E5	ME2
0,000E0	0,000E0	0,000E0	0,000E0	0,000E0	0,000E0	0,000E0	0,000E0	0,000E0	2,152E6	5,940E5	0,000E0	ATXN2L
0,000E0	0,000E0	0,000E0	0,000E0	0,000E0	0,000E0	0,000E0	0,000E0	0,000E0	1,879E6	2,418E6	2,760E5	UBAP2L
0,000E0	0,000E0	0,000E0	4,373E5	0,000E0	0,000E0	0,000E0	0,000E0	0,000E0	4,019E6	5,004E6	2,962E6	PSMD11
0,000E0	0,000E0	0,000E0	0,000E0	0,000E0	0,000E0	0,000E0	0,000E0	0,000E0	0,000E0	1,503E6	1,435E6	NPEPPS
0,000E0	0,000E0	0,000E0	0,000E0	0,000E0	0,000E0	0,000E0	0,000E0	0,000E0	0,000E0	7,058E6	1,208E5	PMPCA
0,000E0	0,000E0	0,000E0	0,000E0	0,000E0	0,000E0	0,000E0	0,000E0	0,000E0	8,368E5	3,268E6	1,082E6	ERBIN
0,000E0	0,000E0	0,000E0	0,000E0	0,000E0	0,000E0	0,000E0	0,000E0	0,000E0	9,596E5	4,712E5	0,000E0	WDR45
0,000E0	0,000E0	0,000E0	0,000E0	0,000E0	0,000E0	0,000E0	0,000E0	0,000E0	3,388E6	3,477E6	2,106E6	DAO
0,000E0	0,000E0	0,000E0	0,000E0	0,000E0	0,000E0	0,000E0	0,000E0	0,000E0	0,000E0	2,764E6	8,845E5	ACP6
0,000E0	0,000E0	0,000E0	0,000E0	0,000E0	0,000E0	0,000E0	0,000E0	0,000E0	1,326E6	5,226E6	4,794E6	UBXN4
0,000E0	0,000E0	0,000E0	0,000E0	0,000E0	0,000E0	0,000E0	0,000E0	0,000E0	0,000E0	4,749E5	2,162E6	ADRM1
2,940E6	0,000E0	0,000E0	0,000E0	0,000E0	0,000E0	0,000E0	1,754E6	2,691E6	3,228E6	7,452E6	4,299E6	EML4
0,000E0	0,000E0	0,000E0	0,000E0	0,000E0	0,000E0	0,000E0	0,000E0	0,000E0	1,605E6	7,983E6	1,963E6	CNDP2
0,000E0	0,000E0	0,000E0	0,000E0	0,000E0	0,000E0	0,000E0	0,000E0	0,000E0	0,000E0	9,528E5	5,245E5	SLC25A13
0,000E0	0,000E0	0,000E0	0,000E0	0,000E0	0,000E0	0,000E0	0,000E0	0,000E0	0,000E0	1,564E6	1,040E6	PDLIM5

0,000E0	0,000E0	0,000E0	0,000E0	0,000E0	0,000E0	0,000E0	0,000E0	0,000E0	0,000E0	1,273E6	6,005E5	CAPNS1
0,000E0	0,000E0	0,000E0	0,000E0	0,000E0	0,000E0	0,000E0	0,000E0	0,000E0	0,000E0	2,461E6	2,637E6	IDH1
7,618E5	0,000E0	0,000E0	0,000E0	0,000E0	0,000E0	0,000E0	0,000E0	1,565E6	2,081E6	1,645E6	1,686E6	ALDH1L1
0,000E0	0,000E0	0,000E0	0,000E0	0,000E0	0,000E0	0,000E0	0,000E0	0,000E0	8,118E5	1,827E6	2,920E6	ALMS1
0,000E0	0,000E0	0,000E0	0,000E0	0,000E0	0,000E0	0,000E0	0,000E0	0,000E0	0,000E0	1,854E6	1,044E6	ECI1
0,000E0	0,000E0	0,000E0	0,000E0	0,000E0	0,000E0	0,000E0	0,000E0	0,000E0	0,000E0	6,831E5	3,264E5	PRDX3
0,000E0	0,000E0	0,000E0	0,000E0	0,000E0	0,000E0	0,000E0	0,000E0	0,000E0	0,000E0	8,070E5	7,512E5	DIABLO
0,000E0	0,000E0	0,000E0	0,000E0	0,000E0	0,000E0	0,000E0	0,000E0	0,000E0	4,719E5	1,684E6	0,000E0	SNX2
0,000E0	0,000E0	0,000E0	0,000E0	0,000E0	0,000E0	0,000E0	0,000E0	0,000E0	1,085E5	3,145E5	0,000E0	-//-
0,000E0	0,000E0	0,000E0	0,000E0	0,000E0	0,000E0	0,000E0	0,000E0	0,000E0	0,000E0	1,754E6	2,448E5	MAIP1
0,000E0	0,000E0	0,000E0	0,000E0	0,000E0	0,000E0	0,000E0	0,000E0	0,000E0	8,598E5	5,077E6	2,912E6	PITRM1
0,000E0	0,000E0	0,000E0	0,000E0	0,000E0	0,000E0	0,000E0	0,000E0	0,000E0	2,613E6	4,919E5	0,000E0	CTNND1
0,000E0	0,000E0	0,000E0	0,000E0	0,000E0	0,000E0	0,000E0	0,000E0	0,000E0	0,000E0	7,194E5	2,020E6	YARS2
0,000E0	0,000E0	0,000E0	0,000E0	0,000E0	0,000E0	0,000E0	0,000E0	0,000E0	1,182E6	1,617E6	1,393E6	DSG2
0,000E0	0,000E0	0,000E0	0,000E0	0,000E0	0,000E0	0,000E0	0,000E0	0,000E0	8,658E5	8,981E5	0,000E0	EIF3E
0,000E0	0,000E0	0,000E0	0,000E0	0,000E0	0,000E0	0,000E0	0,000E0	0,000E0	1,560E6	1,939E6	0,000E0	FUBP3
0,000E0	0,000E0	0,000E0	0,000E0	0,000E0	0,000E0	0,000E0	0,000E0	0,000E0	1,210E6	5,207E6	8,928E5	AHSA1
0,000E0	0,000E0	0,000E0	0,000E0	0,000E0	0,000E0	0,000E0	0,000E0	0,000E0	7,840E5	0,000E0	3,041E5	ARFIP1
0,000E0	0,000E0	0,000E0	0,000E0	0,000E0	0,000E0	0,000E0	0,000E0	0,000E0	0,000E0	2,534E6	3,707E5	MMUT
0,000E0	0,000E0	0,000E0	0,000E0	0,000E0	0,000E0	0,000E0	0,000E0	0,000E0	1,662E6	2,619E6	0,000E0	ATP5F1C
0,000E0	0,000E0	0,000E0	0,000E0	0,000E0	0,000E0	0,000E0	0,000E0	0,000E0	0,000E0	7,154E5	5,542E5	PSMD7
0,000E0	0,000E0	0,000E0	0,000E0	0,000E0	0,000E0	0,000E0	0,000E0	0,000E0	0,000E0	7,650E5	3,598E6	PRKACA
0,000E0	0,000E0	0,000E0	0,000E0	0,000E0	0,000E0	0,000E0	0,000E0	0,000E0	0,000E0	4,236E6	1,324E7	TMX1
0,000E0	0,000E0	0,000E0	0,000E0	0,000E0	0,000E0	0,000E0	0,000E0	0,000E0	0,000E0	5,580E5	2,224E5	UQCRC2
9,840E5	0,000E0	0,000E0	0,000E0	0,000E0	0,000E0	0,000E0	9,654E5	1,021E6	2,731E6	4,554E6	1,120E6	DYNC1H1
0,000E0	0,000E0	0,000E0	0,000E0	0,000E0	0,000E0	0,000E0	0,000E0	0,000E0	2,189E6	5,434E6	5,233E5	ETFDH
0,000E0	0,000E0	0,000E0	0,000E0	0,000E0	0,000E0	0,000E0	0,000E0	0,000E0	2,100E5	1,378E6	3,993E5	TMED9
1,174E6	0,000E0	0,000E0	0,000E0	0,000E0	0,000E0	0,000E0	0,000E0	6,637E5	1,888E6	3,194E6	1,511E6	IQGAP1
0,000E0	0,000E0	0,000E0	0,000E0	0,000E0	0,000E0	0,000E0	0,000E0	0,000E0	2,355E6	1,559E6	3,416E6	MIA3
0,000E0	0,000E0	0,000E0	0,000E0	0,000E0	0,000E0	0,000E0	0,000E0	0,000E0	0,000E0	3,074E6	2,551E6	MAP4
0,000E0	0,000E0	0,000E0	0,000E0	0,000E0	0,000E0	0,000E0	0,000E0	0,000E0	1,223E6	8,817E5	0,000E0	ACOT7
0,000E0	0,000E0	0,000E0	0,000E0	0,000E0	0,000E0	0,000E0	0,000E0	0,000E0	3,591E5	1,351E6	7,417E5	HARS1
0,000E0	0,000E0	0,000E0	0,000E0	0,000E0	0,000E0	0,000E0	0,000E0	0,000E0	5,738E6	1,806E6	2,804E6	PRRC2C
0,000E0	0,000E0	0,000E0	0,000E0	0,000E0	0,000E0	0,000E0	0,000E0	0,000E0	4,911E5	4,784E6	1,442E6	GIGYF2
0,000E0	0,000E0	0,000E0	0,000E0	0,000E0	0,000E0	0,000E0	0,000E0	0,000E0	1,522E6	2,638E6	1,665E6	PGRMC2
0,000E0	0,000E0	0,000E0	0,000E0	0,000E0	0,000E0	0,000E0	0,000E0	0,000E0	0,000E0	1,464E6	1,870E6	LONP1
0,000E0	0,000E0	0,000E0	0,000E0	0,000E0	0,000E0	0,000E0	0,000E0	0,000E0	1,910E6	2,019E6	0,000E0	SMAD3
0,000E0	0,000E0	0,000E0	0,000E0	0,000E0	0,000E0	0,000E0	0,000E0	0,000E0	0,000E0	6,459E5	1,147E6	MBOAT7
3,077E5	0,000E0	0,000E0	0,000E0	7,756E4	0,000E0	0,000E0	0,000E0	0,000E0	3,129E6	2,732E6	2,544E6	MYO1D
0,000E0	0,000E0	0,000E0	0,000E0	0,000E0	0,000E0	0,000E0	0,000E0	0,000E0	6,238E5	1,738E6	0,000E0	TNRC6B
0,000E0	0,000E0	0,000E0	0,000E0	0,000E0	0,000E0	0,000E0	0,000E0	0,000E0	0,000E0	6,719E6	9,750E6	PITHD1
0,000E0	0,000E0	0,000E0	0,000E0	0,000E0	0,000E0	0,000E0	0,000E0	0,000E0	4,434E6	3,647E6	4,625E6	PLEKHA5
0,000E0	0,000E0	0,000E0	0,000E0	0,000E0	0,000E0	0,000E0	0,000E0	0,000E0	0,000E0	7,500E5	2,076E6	SLC3A2
0,000E0	0,000E0	0,000E0	0,000E0	0,000E0	0,000E0	0,000E0	0,000E0	0,000E0	0,000E0	2,101E6	4,856E6	LARP1
0,000E0	0,000E0	0,000E0	0,000E0	0,000E0	0,000E0	0,000E0	0,000E0	0,000E0	1,330E6	8,574E5	9,198E5	UBAP2
0,000E0	0,000E0	0,000E0	0,000E0	0,000E0	0,000E0	0,000E0	0,000E0	0,000E0	0,000E0	1,122E6	2,235E6	CARS1
0,000E0	0,000E0	0,000E0	0,000E0	0,000E0	0,000E0	0,000E0	0,000E0	0,000E0	0,000E0	2,983E6	7,194E5	ADAR
0,000E0	0,000E0	0,000E0	0,000E0	0,000E0	0,000E0	0,000E0	0,000E0	0,000E0	0,000E0	3,808E6	1,322E6	ITPRID2
0,000E0	0,000E0	0,000E0	0,000E0	0,000E0	0,000E0	0,000E0	0,000E0	0,000E0	1,887E6	2,487E6	4,693E6	PGM1
0,000E0	0,000E0	0,000E0	0,000E0	0,000E0	0,000E0	0,000E0	0,000E0	0,000E0	1,532E6	4,035E6	0,000E0	MYO6
0,000E0	0,000E0	0,000E0	0,000E0	0,000E0	0,000E0	0,000E0	0,000E0	0,000E0	1,275E7	0,000E0	1,949E7	FAM120A
0,000E0	0,000E0	0,000E0	0,000E0	0,000E0	0,000E0	0,000E0	0,000E0	4,383E5	8,214E5	8,756E5	5,114E5	STT3A
0,000E0	0,000E0	0,000E0	0,000E0	0,000E0	0,000E0	0,000E0	0,000E0	0,000E0	1,285E6	1,341E6	0,000E0	PPP1R9B
0,000E0	0,000E0	0,000E0	0,000E0	0,000E0	0,000E0	0,000E0	0,000E0	0,000E0	0,000E0	1,000E6	7,569E5	CHD4
0,000E0	0,000E0	0,000E0	0,000E0	0,000E0	0,000E0	0,000E0	0,000E0	0,000E0	0,000E0	1,623E6	2,032E6	ALDH5A1
0,000E0	0,000E0	0,000E0	0,000E0	0,000E0	0,000E0	0,000E0	0,000E0	0,000E0	3,558E5	2,863E6	3,982E6	PPP6R3

0,000E0	0,000E0	0,000E0	0,000E0	0,000E0	0,000E0	0,000E0	0,000E0	0,000E0	7,945E5	1,594E6	7,685E5	SF3B3
0,000E0	0,000E0	0,000E0	0,000E0	0,000E0	0,000E0	0,000E0	0,000E0	0,000E0	6,430E5	0,000E0	8,715E5	MAT2A
0,000E0	0,000E0	0,000E0	0,000E0	0,000E0	0,000E0	0,000E0	0,000E0	0,000E0	0,000E0	1,627E6	1,605E6	ZFR
0,000E0	0,000E0	0,000E0	0,000E0	0,000E0	0,000E0	0,000E0	0,000E0	0,000E0	0,000E0	5,833E5	1,583E6	FKBP5
0,000E0	0,000E0	0,000E0	0,000E0	0,000E0	0,000E0	0,000E0	0,000E0	0,000E0	1,768E6	4,081E6	0,000E0	ESYT1
0,000E0	0,000E0	0,000E0	0,000E0	0,000E0	0,000E0	0,000E0	0,000E0	0,000E0	1,983E6	3,766E6	4,276E6	ACOT9
0,000E0	0,000E0	0,000E0	0,000E0	0,000E0	0,000E0	0,000E0	0,000E0	0,000E0	1,157E7	2,769E6	3,951E6	MCM4
0,000E0	0,000E0	0,000E0	0,000E0	0,000E0	0,000E0	0,000E0	0,000E0	0,000E0	1,372E6	1,534E6	3,343E6	UTRN
0,000E0	0,000E0	0,000E0	0,000E0	0,000E0	0,000E0	0,000E0	0,000E0	0,000E0	1,137E6	0,000E0	3,956E5	PPFIBP1
0,000E0	0,000E0	0,000E0	0,000E0	0,000E0	0,000E0	0,000E0	0,000E0	0,000E0	1,353E6	2,454E6	4,956E6	NUMA1
0,000E0	0,000E0	0,000E0	0,000E0	0,000E0	0,000E0	0,000E0	0,000E0	0,000E0	2,534E5	0,000E0	3,853E5	HK2
0,000E0	0,000E0	0,000E0	0,000E0	0,000E0	0,000E0	0,000E0	0,000E0	0,000E0	0,000E0	1,422E6	8,861E5	FAM83H
0,000E0	0,000E0	0,000E0	0,000E0	0,000E0	0,000E0	0,000E0	0,000E0	0,000E0	5,166E5	1,002E6	0,000E0	DDX18
0,000E0	0,000E0	0,000E0	0,000E0	0,000E0	0,000E0	0,000E0	0,000E0	0,000E0	1,515E6	3,606E6	3,343E6	PLEKHG3
0,000E0	0,000E0	0,000E0	0,000E0	0,000E0	0,000E0	0,000E0	0,000E0	0,000E0	0,000E0	7,496E5	1,137E6	MIPEP
0,000E0	0,000E0	0,000E0	0,000E0	0,000E0	0,000E0	0,000E0	0,000E0	0,000E0	0,000E0	8,664E5	9,035E5	GLG1
0,000E0	0,000E0	0,000E0	0,000E0	0,000E0	0,000E0	0,000E0	0,000E0	0,000E0	2,268E6	4,140E6	3,399E6	TJP2
0,000E0	0,000E0	0,000E0	0,000E0	0,000E0	0,000E0	0,000E0	0,000E0	0,000E0	6,285E5	2,617E6	3,015E6	COBLL1
0,000E0	0,000E0	0,000E0	0,000E0	0,000E0	0,000E0	0,000E0	0,000E0	0,000E0	2,100E5	0,000E0	9,523E5	PDE4DIP
0,000E0	0,000E0	0,000E0	0,000E0	0,000E0	0,000E0	0,000E0	0,000E0	0,000E0	2,937E6	5,323E6	0,000E0	GCN1

For AmotL2 p100 binding positive partners:

1) ‘p100+biotin’ samples > ‘p100-biotin’ samples (average)
2) ‘p100+biotin’ samples > all ctrl samples (average)
3) minium of ‘p100+biotin’ samples ≥ maxium of ‘ctrl’ sample
4) at least 2/3 ‘p100+biotin’ samples >0
