# Supplementary material for: Deciphering the Role of p60AmotL2 in Epithelial Extrusion and Cell Detachment
Source: Cells. 2023 Aug 28;12(17):2158. doi: 10.3390/cells12172158 (PMC10486482; doi:10.3390/cells12172158)
Supplement: Supplementary file 1 [file cells-12-02158-s001.zip › Table S2. KEGG pathway analysis of AmotL2 BioID-MS data in MDCK cells. (1).pdf]

Supplementary Table. KEGG pathway analysis of AmotL2 BioID-MS data in MDCK cells.

term	p-value	adjusted P-value	-log 10 adjusted P-value	genes
Adherens junction	4,3116E-05	0,007031	2,152955929	TJP1, SMAD3, CTNND1, CTNNA1, IQGAP1, VCL
Bacterial invasion of epithelial cells	6,8266E-05	0,007031	2,152955929	CTTN, ARPC2, CLTC, CTNNA1, CRK, VCL
Pentose phosphate pathway	0,00014481	0,00838	2,076734738	GPI, G6PD, PGM1, PFKP
Protein processing in endoplasmic reticulum	0,00016273	0,00838	2,076734738	SSR4, DAD1, RPN1, CANX, STT3A, SEC61B, UBXN4, CKAP4
SNARE interactions in vesicular transport	0,00021173	0,008723	2,059327528	STX7, SEC22B, VAMP5, VAMP3
Aminoacyl-tRNA biosynthesis	0,00032016	0,010108	1,995329657	HARS1, KARS1, CARS1, YARS2, WARS1
Glycolysis / Gluconeogenesis	0,00034348	0,010108	1,995329657	GPI, HK2, PGM1, PFKP, PCK2
Central carbon metabolism in cancer	0,00042121	0,010846	1,964728641	G6PD, IDH1, HK2, PFKP, GLS
Proteasome	0,0007709	0,017645	1,753378439	PSMD11, PSMD7, PSME3, ADRM1
Tight junction	0,00086299	0,017778	1,75012887	TJP1, PATJ, ARPC2, CTTN, MYH9, PRKACA, TJP2

186 proteins listed in Supplementary Table
Top 10 enrtiched pathways
Statistical information
http://amp.pharm.mssm.edu/Enrichr
