# Supplementary figures and images for: Deciphering the Role of p60AmotL2 in Epithelial Extrusion and Cell Detachment
Source: Cells. 2023 Aug 28;12(17):2158. doi: 10.3390/cells12172158 (PMC10486482; doi:10.3390/cells12172158)

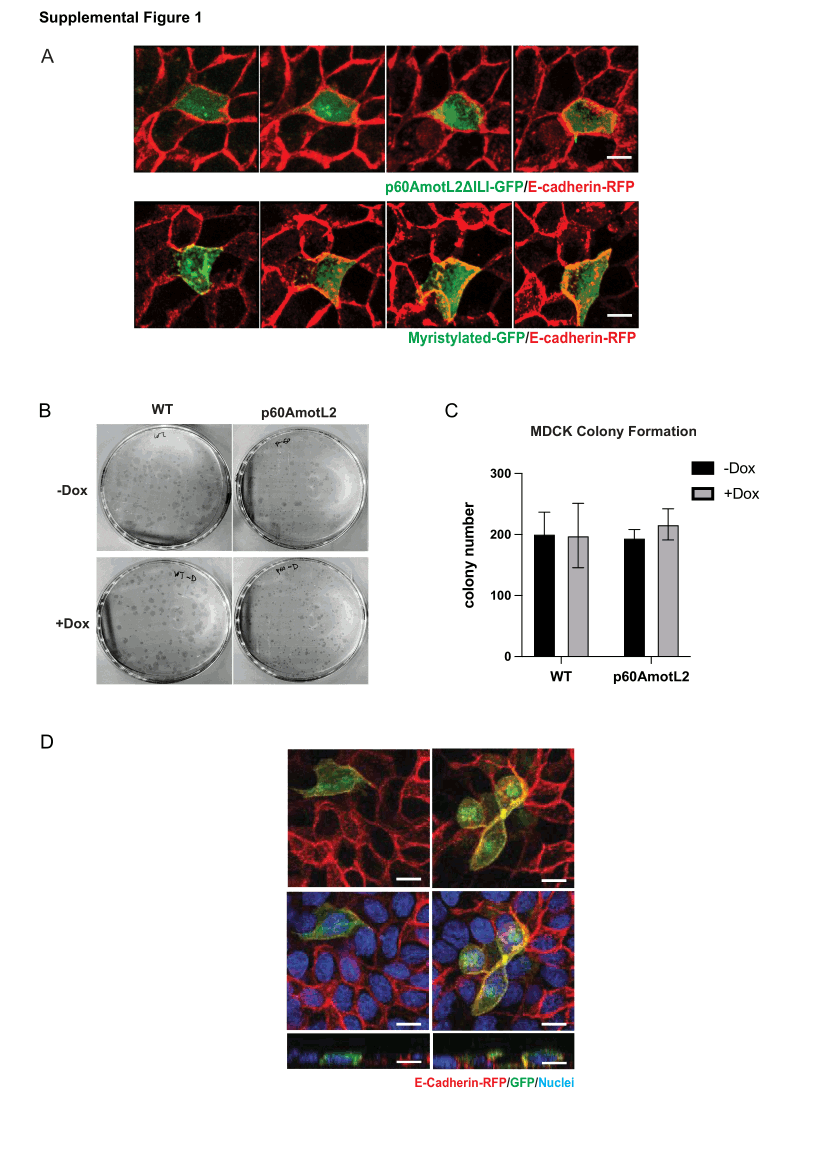

Supplement: Supplementary file 1 [file cells-12-02158-s001.zip › Supplemental Figure S1.gif]

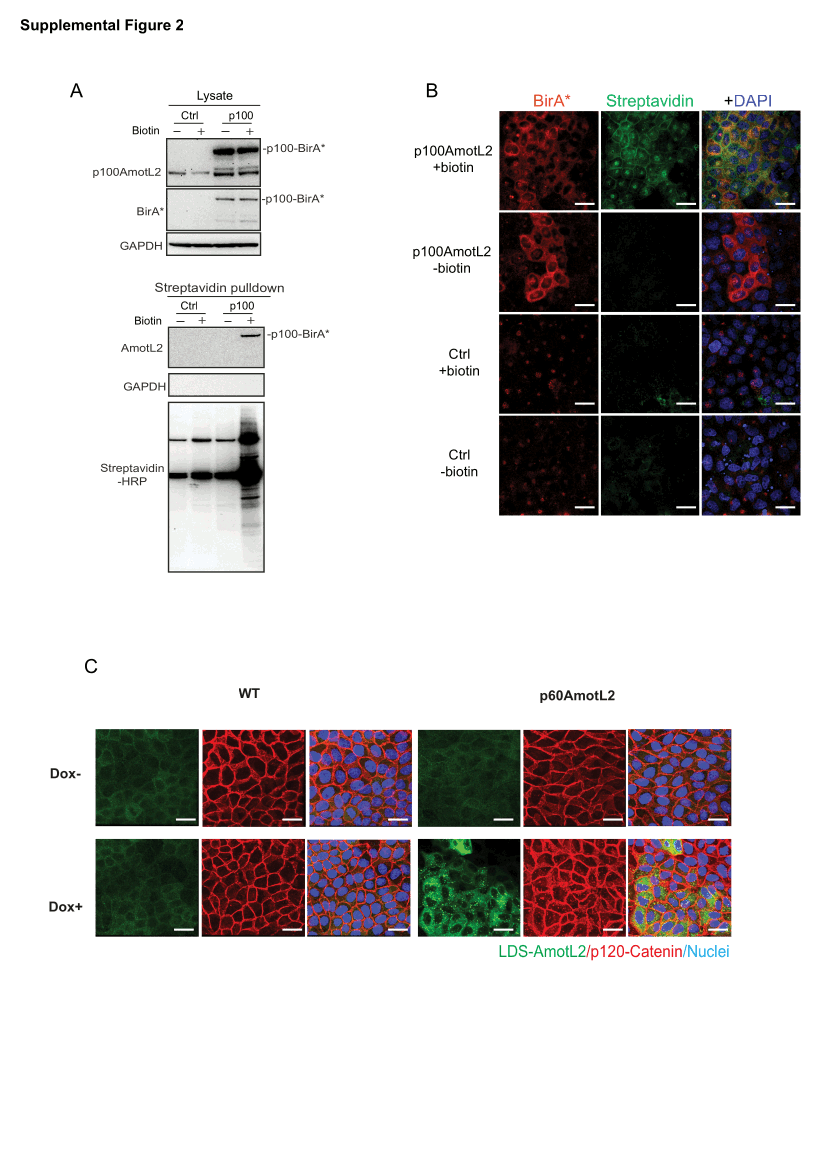

Supplement: Supplementary file 1 [file cells-12-02158-s001.zip › Supplemental figure S2.gif]

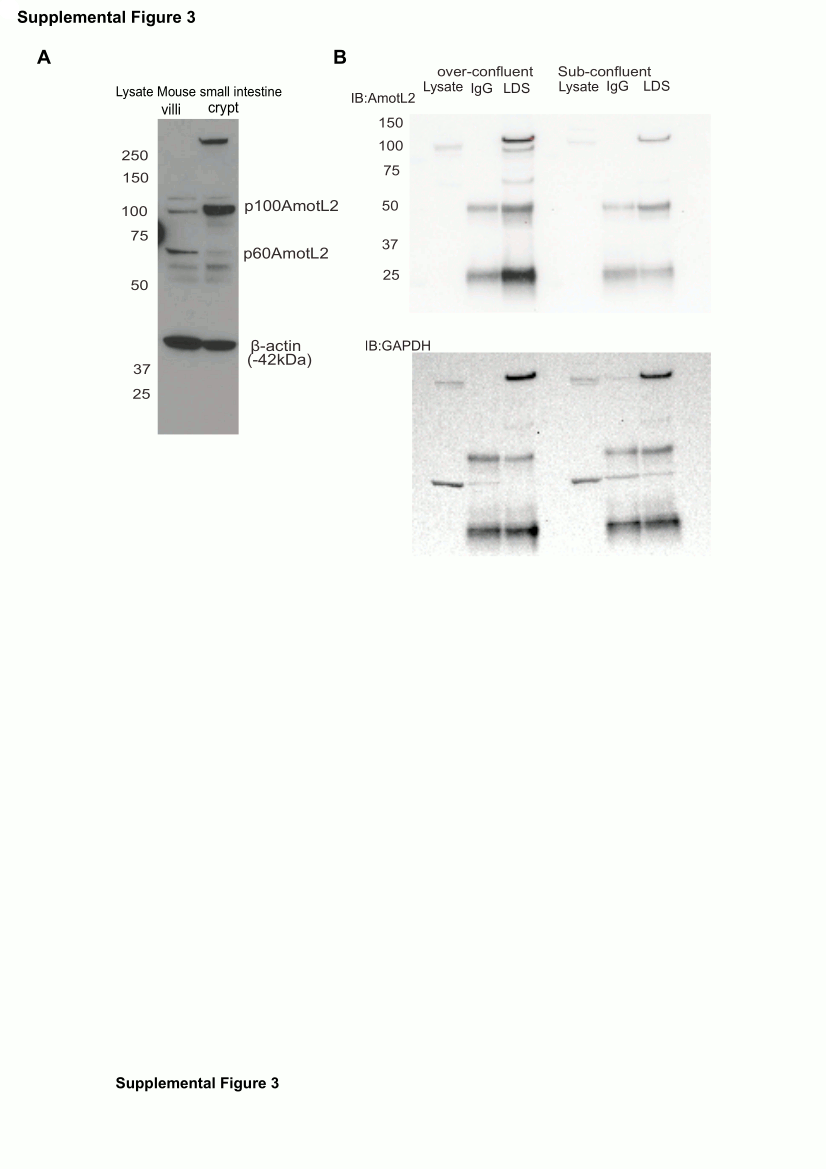

Supplement: Supplementary file 1 [file cells-12-02158-s001.zip › supplemental figure S3.gif]

## Slide 1
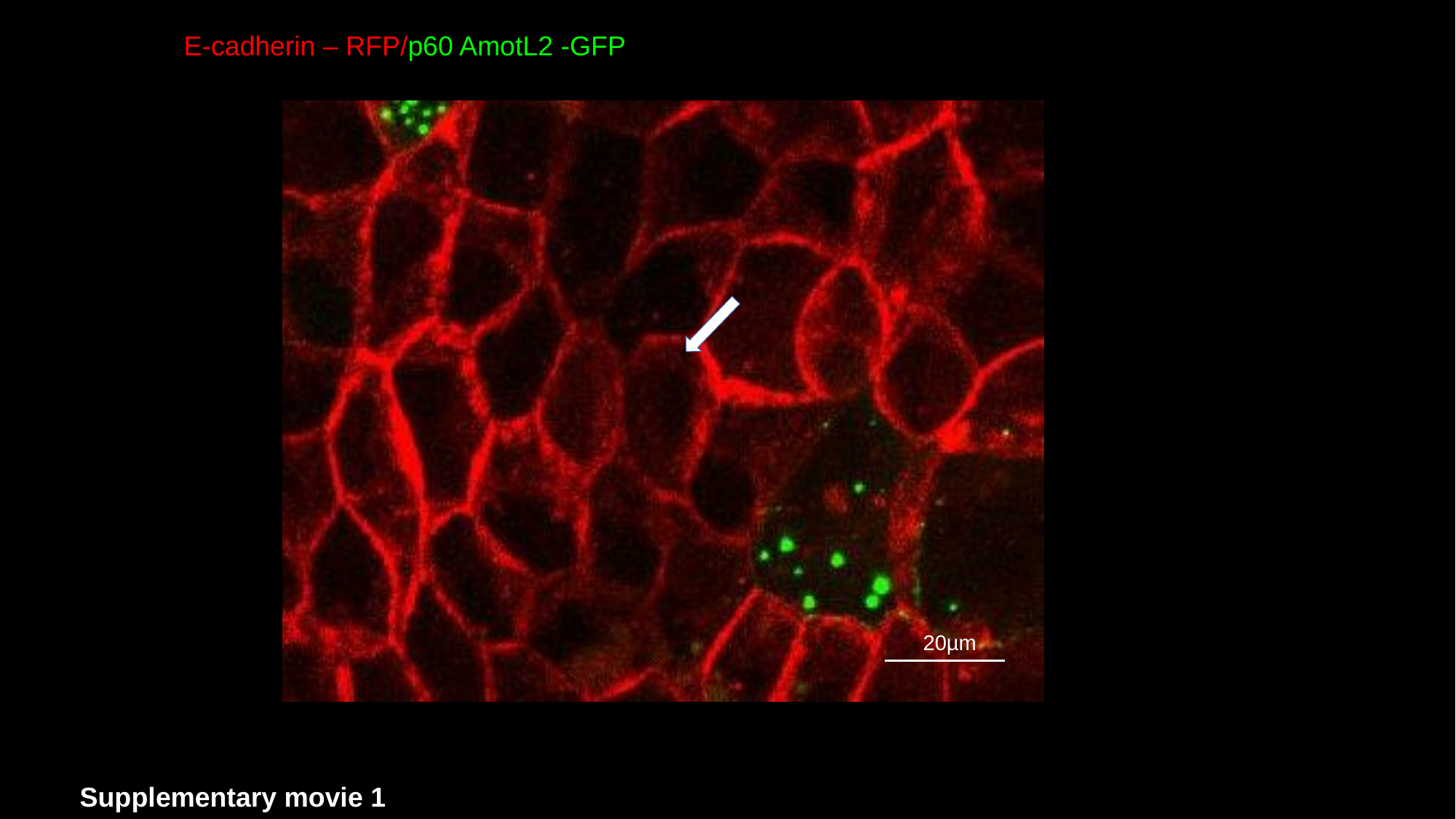

E-cadherin – RFP/p60 AmotL2 -GFP
20µm
Supplementary movie 1

Supplement: Supplementary file 1 [file cells-12-02158-s001.zip › supplementary movie S1.pptx]

## Slide 1
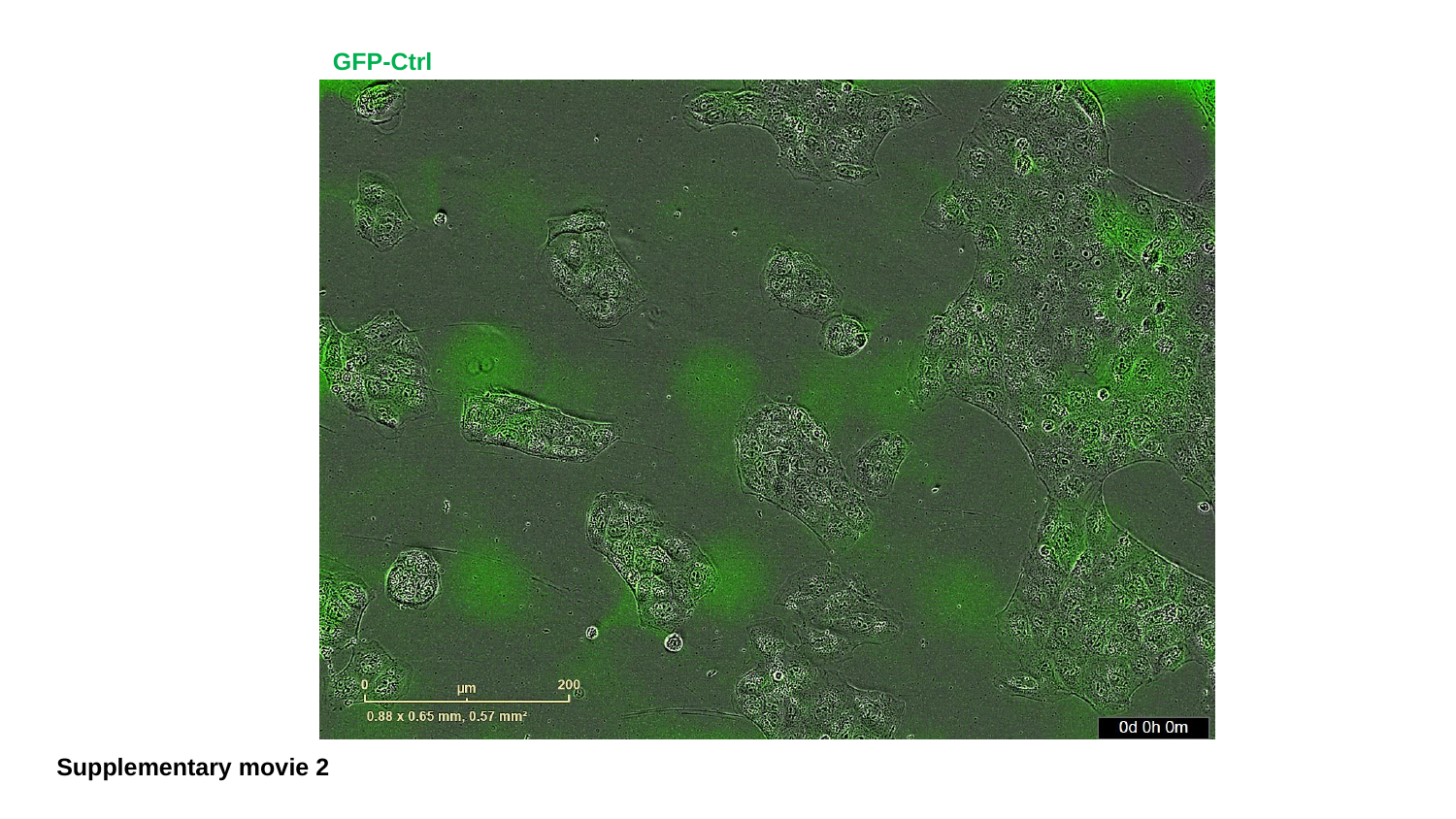

GFP-Ctrl
Supplementary movie 2

Supplement: Supplementary file 1 [file cells-12-02158-s001.zip › Supplementary movie S2.pptx]

## Slide 1
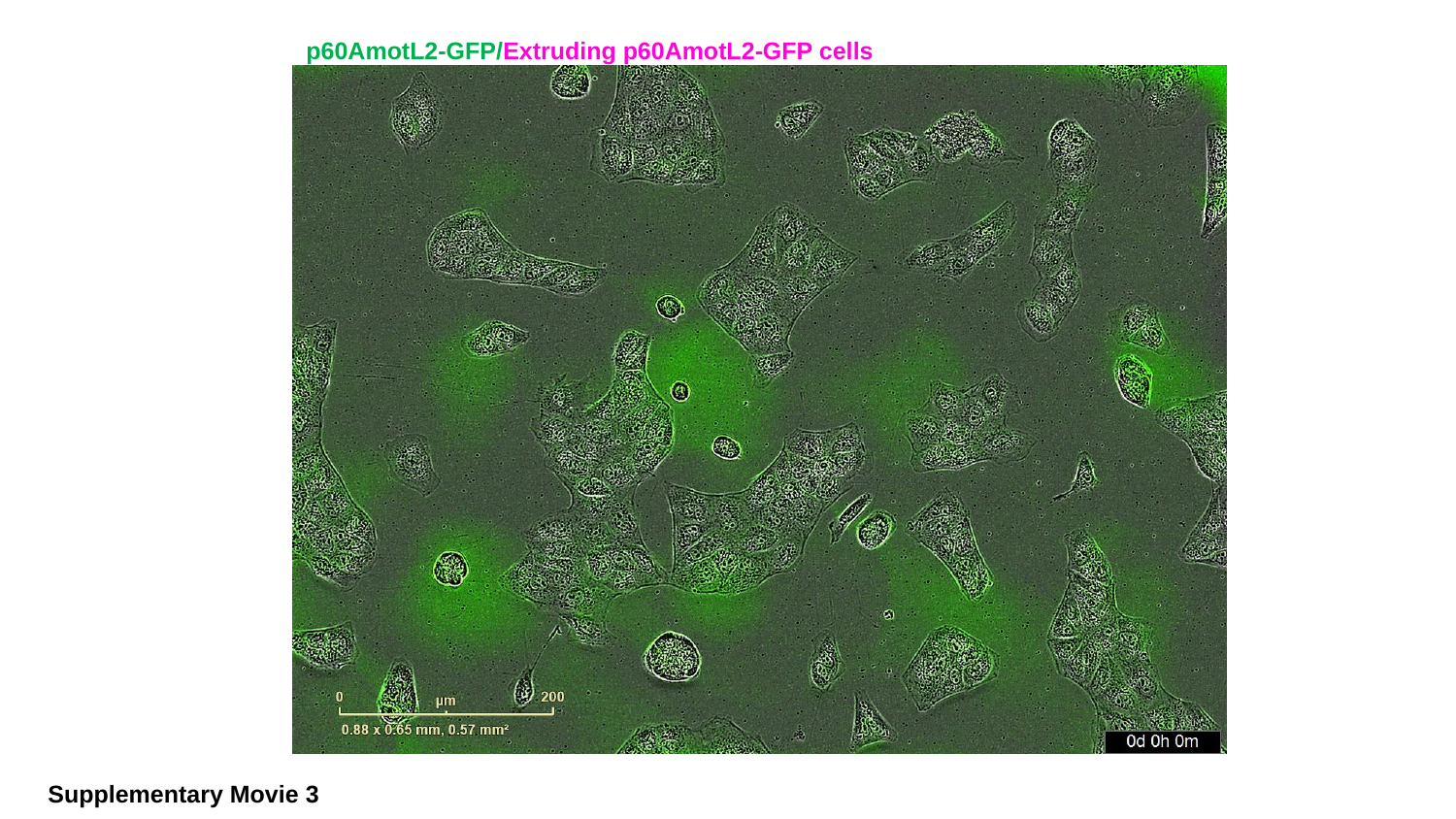

p60AmotL2-GFP/Extruding p60AmotL2-GFP cells
Supplementary Movie 3

Supplement: Supplementary file 1 [file cells-12-02158-s001.zip › Supplementary movie S3.pptx]

## Slide 1
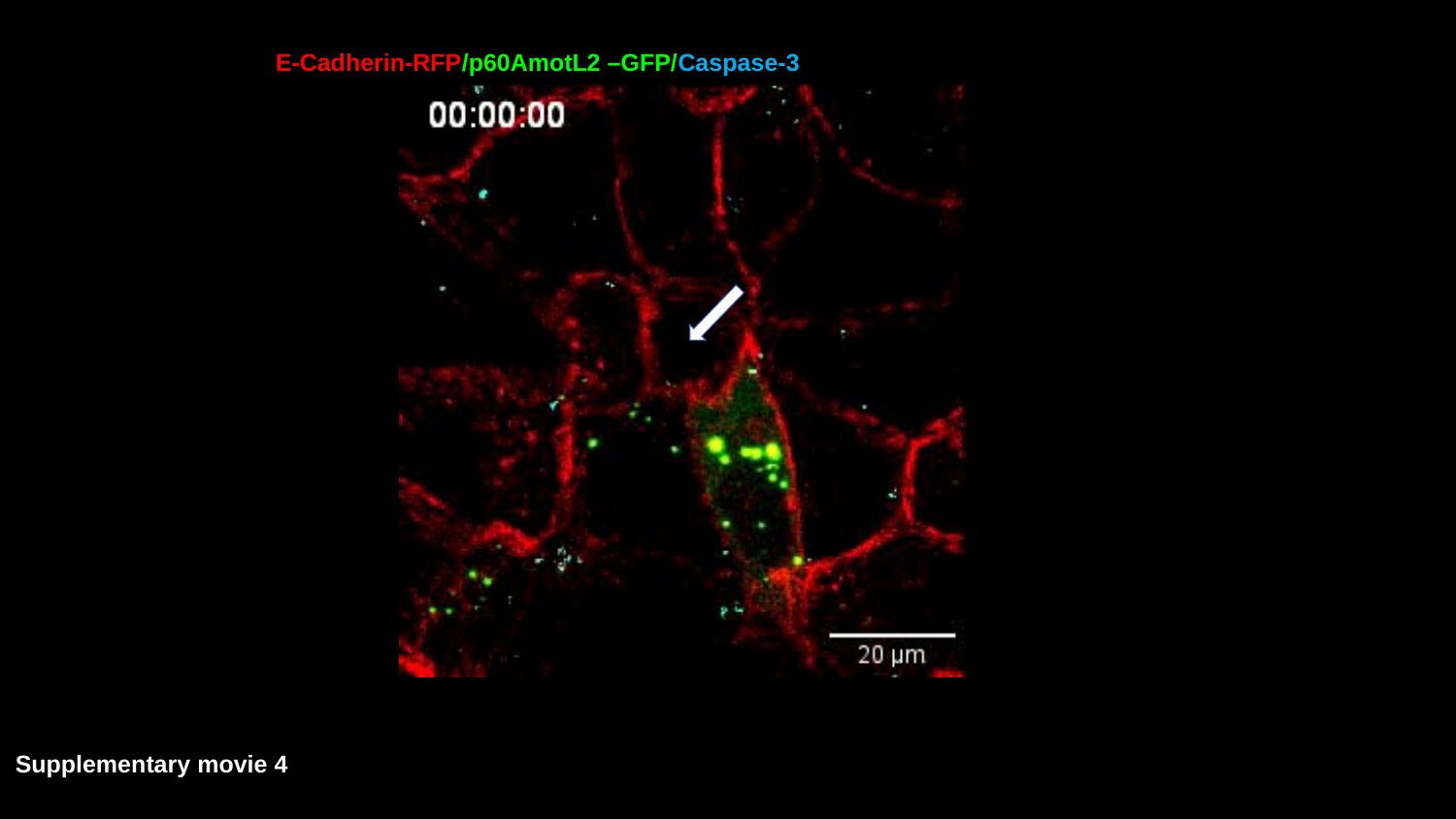

E-Cadherin-RFP/p60AmotL2 –GFP/Caspase-3
Supplementary movie 4

Supplement: Supplementary file 1 [file cells-12-02158-s001.zip › Supplementary movie S4.pptx]
